# Choreography of the Transcriptome, Photophysiology, and Cell Cycle of a Minimal Photoautotroph, *Prochlorococcus*

**DOI:** 10.1371/journal.pone.0005135

**Published:** 2009-04-08

**Authors:** Erik R. Zinser, Debbie Lindell, Zackary I. Johnson, Matthias E. Futschik, Claudia Steglich, Maureen L. Coleman, Matthew A. Wright, Trent Rector, Robert Steen, Nathan McNulty, Luke R. Thompson, Sallie W. Chisholm

**Affiliations:** 1 Department of Civil and Environmental Engineering, Massachusetts Institute of Technology, Cambridge, Massachusetts, United States of America; 2 Department of Microbiology, University of Tennessee, Knoxville, Tennessee, United States of America; 3 Faculty of Biology, Technion-Israel Institute of Technology, Haifa, Israel; 4 Department of Oceanography, University of Hawaii, Honolulu, Hawaii, United States of America; 5 Institute of Theoretical Biology, Humboldt University, Berlin, Germany; 6 Center for Molecular and Structural Biomedicine, University of Algarve, Faro, Portugal; 7 Institute of Biology III, University of Freiburg, Freiburg, Germany; 8 Department of Genetics, Harvard Medical School, Boston, Massachusetts, United States of America; 9 Department of Biology, Massachusetts Institute of Technology, Cambridge, Massachusetts, United States of America; Universidad Miguel Hernandez, Spain

## Abstract

The marine cyanobacterium *Prochlorococcus* MED4 has the smallest genome and cell size of all known photosynthetic organisms. Like all phototrophs at temperate latitudes, it experiences predictable daily variation in available light energy which leads to temporal regulation and partitioning of key cellular processes. To better understand the tempo and choreography of this minimal phototroph, we studied the entire transcriptome of the cell over a simulated daily light-dark cycle, and placed it in the context of diagnostic physiological and cell cycle parameters. All cells in the culture progressed through their cell cycles in synchrony, thus ensuring that our measurements reflected the behavior of individual cells. Ninety percent of the annotated genes were expressed, and 80% had cyclic expression over the diel cycle. For most genes, expression peaked near sunrise or sunset, although more subtle phasing of gene expression was also evident. Periodicities of the transcripts of genes involved in physiological processes such as in cell cycle progression, photosynthesis, and phosphorus metabolism tracked the timing of these activities relative to the light-dark cycle. Furthermore, the transitions between photosynthesis during the day and catabolic consumption of energy reserves at night— metabolic processes that share some of the same enzymes — appear to be tightly choreographed at the level of RNA expression. In-depth investigation of these patterns identified potential regulatory proteins involved in balancing these opposing pathways. Finally, while this analysis has not helped resolve how a cell with so little regulatory capacity, and a ‘deficient’ circadian mechanism, aligns its cell cycle and metabolism so tightly to a light-dark cycle, it does provide us with a valuable framework upon which to build when the *Prochlorococcus* proteome and metabolome become available.

## Introduction

The unicellular cyanobacterium *Prochlorococcus* is believed to be the most abundant photosynthetic organism on Earth [1]. It is also the smallest oxygenic phototroph, both in physical size (0.6 microns in diameter) and genome size. The latter ranges from 1.64–2.68 Mbp in a set of strains that span the currently known phylogenetic diversity of this group [2]. The streamlined genome appears to be accompanied by a reduction in regulatory capacity. Strain MED4, for example, contains only five sigma factors, five sensor histidine kinases, and seven response regulators, considerably fewer than that found in other bacteria [3]. The relative number of non-coding RNAs is comparable to that found in other bacteria, however [4], which suggests an unusual regulation portfolio in this organism. Rapid shifts in temperature, salinity, pH, and other physical variables are rare in the ocean environment, and nutrients are typically maintained at extremely low concentrations, except during deep mixing events in seasonal environments. The overall reduction in regulatory capacity could be viewed as streamlining for life in a relatively static environment.

Life in the nutrient-poor open ocean is not devoid of dynamism, however. Sunlight, the energy source for *Prochlorococcus*, undergoes a regular and dramatic variation in supply each day. It is not surprising, therefore, to find that cellular metabolism has been shaped by this diel energy flux. Carbon fixation in *Prochlorococcus* has been shown to occur exclusively during the day, with approximately 2/3 of the total carbon accumulation occurring before mid-day [5], [6]. Other photosynthetic parameters, such as photochemical efficiency of photosystem II (F_v_/F_m_), quantum yield of chlorophyll fluorescence, maximum quantum yield of carbon fixation, and concentration of the carotenoid accessory pigment zeaxanthin, also showed strong diel variation in prior studies [5], [6].

The expression of a number of photosynthesis genes are known to display periodicity over a diel light/dark cycle in *Prochlorococcus*. Transcripts of genes encoding photosystem II's D1 (*psbA*), D2 (*psbD*), and CP43 (*psbC*), for example, peak in abundance at subjective mid-day, while the major light-harvesting complex (*pcbA*, or *pcb* in strain MED4) has two maxima, one at sunrise, and one at sunset [7]. Expression of the *rbcL* gene encoding the large subunit of the Rubisco, parallels strongly with the carbon fixation rate and maximum quantum yield of carbon fixation, exhibiting a pronounced maximum at sunrise and a dramatic decrease in the afternoon [5], [8].

The cell cycles of *Prochlorococcus* cells cultured on light dark cycles are tightly synchronized [9]–[12]. In populations with mean generation times of one day or longer, which is typical under most conditions [11], [13], DNA synthesis occurs during the afternoon, and cell division — in those cells that divide — occurs only in the late afternoon or early evening [11], [14], [15]. In cases where populations double more than once per day, the second round of division takes place within hours of the first [13]. Not surprisingly, expression of genes involved in initiating cell division (*ftsZ* ) and DNA replication (*dnaA*) varies significantly over the light/dark cycle in synchronized cultures, and are maximal during the S phase [16].

Given the tight cell cycle synchrony on light/dark cycles, and periodicity of so many other cellular functions in *Prochlorococcus*, one might suspect that these processes are regulated through coupling to a circadian oscillator, as is typical of other cyanobacteria. For example, transcription of much of the genome in freshwater cyanobacteria, and the regulation of key physiological processes in freshwater and marine cyanobacteria, have been found to be under the control of a circadian clock [17]–[21]. Three components, KaiA, KaiB, and KaiC, are necessary and sufficient for the clock to function [21], [22], and transmission of the clock signal to the genome is believed to occur through the SasA-RpaA two-component regulatory system [23] or SasA-independent changes in DNA topology [21], [24]. Light-dependent entrainment of the clock appears to work through CikA, which modifies the phosphorylation state of KaiC [25].

While *Prochlorococcus* contains the clock genes *kaiB* and *kaiC* [12], and they have periodic expression on a light/dark cycle [12], it lacks *kaiA*. The latter is believed to be an essential component of the cyanobacterial clock as it is involved in phosphorylating KaiC, and in helping the clock keep time in absence of light-dark cues. Importantly, whereas cyanobacteria that contain *kaiA* maintain periodic expression under constant light conditions, *Prochlorococcus* does not [12]. Furthermore, several key regulators of the clock that are involved in light-dark entrainment (e.g. CikA) are missing in *Prochlorococcus* [12], suggesting either that *Prochlorococcus* does not have a clock, or that it functions in a different way.

The extremely tight synchrony of cell division in *Prochlorococcus* when grown on a light/dark cycle, its streamlined genome, and its apparent limitations *vis a vis* a functioning circadian oscillator, motivated us to undertake an in-depth analysis of the coordination of the transcriptome, cell cycle, and photophysiology in this cell. The questions driving our study were as follows: What fraction of the entire genome is expressed under optimal growth conditions on a light-dark cycle, and what fraction of those expressed genes are periodic? What is the temporal relationship between the timing of transcription of key genes, and the physiological processes they are associated with? What genes are transcribed at similar times in the cycle, and does this clustering tell us anything about metabolic partitioning? Finally, what can we learn about the global regulation of diel periodicity in gene expression, particularly as this cell seems to lack a circadian clock?

## Results and Discussion

**Figure 1:**
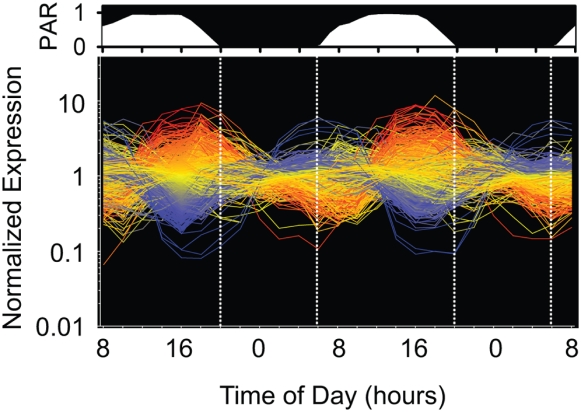
Relative RMA-normalized expression levels of all annotated open reading frames in MED4 over a two-day diel. Relative PAR (photosynthetically available radiation) over the experiment is represented above the expression patterns. Each line represents one of the 1698 unique open reading frames (line colors are arbitrary). Vertical dotted lines denote light/dark transitions.

*Prochlorococcus* strain MED4, a member of the high-light adapted clade of *Prochlorococcus* that dominates surface waters over much of the mid-latitude oceans [26] was used for this study. It has one of the smallest genomes of all cultured *Prochlorococcus* strains, synchronizes tightly to a light dark cycle, and can achieve a growth rate of one doubling per day under optimal conditions. The doubling times of the replicate cultures used in this study were 1.1 and 1.0 days, and thus the cells within the population progressed through the cell cycle in synchrony. The important consequence is that our population-level measurements of gene expression and cell physiology approximate what is happening in an individual cell. As a result, the periodicity in the global transcriptome was very well defined and reproducible over both days of sampling in both of the replicate cultures (Figure 1, and see Table S1 for expression data), forming a solid database for all of our analyses.

### General features of the transcriptome and its response to the light dark cycle

Overall, 89% of the total 1698 analyzed protein-coding genes in this cell were expressed at detectable levels (see Materials and Methods section) over the photocycle. The remaining genes include 27 that have been shown to be upregulated in response to nutrient and light stress, as well as phage infection [27]–[30] —just a few of the stressors that *Prochlorococcus* cells are likely to experience in the oceans. We hypothesize that the remaining genes with undetectable expression may play similar roles. All *Prochlorococcus* strains sequenced to date share 1273 gene clusters, constituting a well-defined set of ‘core’ genes for this group, which is also supported by analyses of metagenomic databases [2], [31]–[33]. *Prochlorococcus* MED4 contains an additional 615 so-called ‘flexible’ gene clusters, which are found in some, but not all strains of *Prochlorococcus*. Flexible genes are often located in hypervariable genomic islands thought to play a role in adaptation to specific environments. Since the core genes encode basic metabolic processes [2], [34] whereas the ‘flexible’ genes are more specialized, one might expect that the ‘core’ genes would be disproportionately expressed relative to the flexible genes under the optimal growth conditions of our experiments. We found a marginal difference: 91% of the 1288 core genes compared with 83% of the 410 flexible genes were expressed.

Qualitative inspection shows that most of the genes display periodic expression, with a single maximum and minimum per 24 hour photoperiod (Figure 1). Fourier analysis revealed that 91% (with a false discovery rate (FDR) of less than 0.1) of the expressed protein-encoding genes exhibited significant periodicity. In contrast to the protein coding genes, only 68% of non-coding RNAs (excluding tRNA and ribosomal RNA genes) and 67% of antisense RNAs were periodic. Many of the aperiodic ncRNAs are “house keeping” genes such as *rnpB*, *ffs* and *ssrA* (Table S1). Probes derived from the intergenic regions displayed a considerably lower percentage of periodic expression (31%). The intergenic probe sets that exhibited periodicity may correspond to 5′/3′ untranslated regions, genes missed in the initial genome annotation, or short functional RNAs [4]. Of the “flexible” genes, 90% of those expressed were periodic, including those in genomic islands. Thus at the transcriptional level, the flexible genome, and even genomic islands, have similar characteristics as the core genome, lending support to the hypothesis that the flexible genome and genomic islands are physiologically important.

We next looked at the overall features and timing of the expression patterns of the periodically expressed genes. For most genes, peak expression was at the onset of either subjective sunrise or sunset (Figure 1). Quantitative analyses (see Methods) confirmed that the distribution of the time of maximum RNA abundance over the photoperiod for all of the periodic genes was largely bimodal, with most genes peaking in expression within a few hours of subjective sunrise (06:00) or sunset (20:00) (Figure S1). Despite this clustering around dawn and dusk, every hour in the 24 hour photoperiod was the time of peak expression of at least a few genes (Figure S1). To identify the predominant patterns of diel periodicity, we performed “soft clustering” analysis (see Materials and Methods) of the transcriptome. Sixteen clusters of genes could be identified as having similar transcriptome periodicities (Table 1, Figure S2). The size of the clusters ranged from 22 to 138 (average 88) genes and peak transcription levels of the clusters were spread fairly evenly over the photocycle, with the exception of clusters 12 and 13, and 14 and 15, which had peak expression times less than one half hour apart. The gene content of these clusters and their relationships form the heart of the analysis of transcriptome coordination presented below.

**Table 1:** Characteristics of the gene clusters found to be periodic (1–16), aperiodic (17), and non-expressed (18), showing the time of their peak expression (note that h = 0, is 4 hours after the onset of dark in a 14:10 light-dark cycle), and the subcategory of genes enriched in each cluster.

Cluster	# Genes	Mean Fourier Score	Mean peak time (h)	Cyanobase Subcategories enriched	Subcategory genes: Enriched / Total	Enrichment FDR
1	57	15.69	8.3±0.7	8.5 Photosystem I	9/22	1.50E-09
				8.6 Photosystem II	8/22	2.50E-05
2	52	14.7	9.6±0.8			
3	23	15.02	12.5±0.8	8.3 Cytochrome b6/f	3/7	0.0059
				8.6 Photosystem II	8/22	3.70E-08
4	62	14.97	15.8±0.7	8.3 Cytochrome b6/f	3/7	0.073
5	120	15.96	17.5±0.4	8.9 Respiratory terminal oxidases	3/3	0.019
				13.3 Degradation of proteins, peptides, and glycopeptides	5/15	0.071
6	138	15.06	18.6±0.5	9.2 Purine ribonucleotide biosynthesis	7/18	0.0049
7	121	15.54	20.1±0.4	5.2 Nitrogen metabolism	4/8	0.087
8	90	13.97	21.0±0.6	4.3 Chaperones	7/14	0.00023
9	99	13.94	22.4±0.5	12.2 RNA synthesis, modification, and DNA transcription	6/23	0.035
10	77	13.47	0.3±0.6			
11	91	13.71	1.6±0.4			
12	111	13.96	3.0±0.5			
13	110	15.23	3.4±0.4	13.2 Ribosomal proteins	45/53	3.70E-41
14	22	12.95	4.6±0.8			
15	107	14.76	4.7±0.5			
16	125	15.97	5.5±0.5	8.1 ATP synthase	8/8	8.40E-08
				8.2 CO2 metabolism	7/9	1.80E-05
17	173	7.76	N/A			
18	180	8.08	N/A	2.6 Menaquinone and ubiquinone	6/9	0.00045

The false discovery rate (FDR) of the enrichment is also shown.

### Cell growth and the cell division cycle

**Figure 2:**
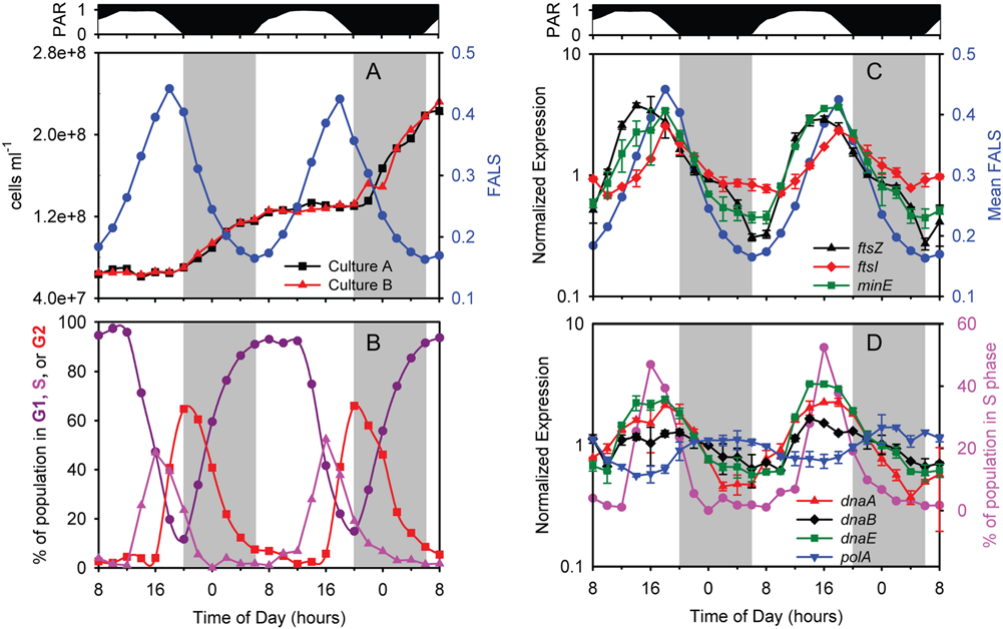
Cell cycle properties of the MED4 cultures during the experiment. (A) Cell abundance for the two individual cultures and mean forward angle light scattering (FALS), a surrogate for cell size, and (B) percentage of cells in G1, S (DNA synthesis), or G2 phase at each time point are shown. Expression time course of (C) cell division genes *ftsZ*, *ftsI*, *minE* and (D) DNA replication genes *dnaA*, *dnaB*, *dnaE*, and *polA*. Mean FALS (C, blue circles) or the percent of the population in S phase (D, pink circles) is shown for comparison. Error bars represent one standard deviation of the mean for the replicate cultures.

The tight synchrony of the cells in the cultures was reflected in a number of the measured variables. The growth of individual cells (as measured by forward light scatter, a proxy for size) began at dawn, and ended two hours before dark, when the cells began to divide (Figure 2A). Cell number increased in the cultures over the dark period, such that all of the cells had divided by sunrise, i.e. the culture had doubled. DNA synthesis (S phase) began approximately six hours after dawn and was complete by the middle of the night (Figure 2B). The G1 and G2 phases of the cell cycle lead and followed the S phase, with some overlap, but on the whole the population displayed remarkable and reproducible synchrony, both between the replicate cultures and over sequential 24 hour periods.

The temporal specificity of DNA synthesis and cell division during the photocycle was matched by the expression of the genes responsible for these activities. MED4 lacks orthologs to most of the 15 protein components of the cell division machinery (“divisome”) of *E. coli* [35], but those it does have were in general maximally expressed prior to the onset of septation (Figure 2C, Table S2). Transcript levels of *ftsZ*, for example, which encodes the cytoplasmic septal Z ring, peaked 4 hours before sunset at the time of the S-phase maximum (Figure 2C), consistent with prior studies [16]. A trio of proteins, MinC, MinD, and MinE, function to establish the location of FtsZ ring formation [36], thus it is not surprising that transcript abundance for *minD* (Table S2) and *minE* (Figure 2C and Table S2) exhibited strong periodicity with a pattern similar to *ftsZ* in our experiment (Figure 2C). The pattern for *minC* was also periodic, though not as strong (Table S2). Expression of *ftsI* and *ftsW*, which together synthesize the septal peptidoglycan once recruited to the Z-ring [35], peaked 1–2 hours after *ftsZ* (Figure 2C and Table S2), timing that is consistent with that of *E. coli* [35]. In contrast, the two paralogs of *ftsI* and *ftsW*, *pbp2* and *rodA* respectively, were expressed aperiodically (Table S2), which is consistent with their function in the synthesis of the cell wall during cell growth rather than division [35]. Two other predicted members of the cell division apparatus, *mraW* and *amiC* - encoding an S-adenosyl-methionine-dependent methyltransferase and a periplasmic amidase, respectively [35] - had undetectable expression or peak expression at 03:00, respectively (Table S2), leaving their role unclear.

As DNA synthesis occurred at a discrete period in the light/dark cycle, so did the peak abundance of the genes involved in this process. Initiation of chromosomal replication involves proteins DnaA and DnaB (helicase), thus it is not surprising that their transcripts accumulated 2–6 hours prior to the onset of DNA replication, and were maximally-abundant at the peak of S phase (Figure 2D and Table S3), confirming prior isolated studies of *dnaA* expression in *Prochlorococcus* [16]. Genes involved in initiation as well as elongation phases of DNA polymerization were likewise maximally abundant during the S phase. This includes 4 out of the 5 genes encoding DNA polymerase III (e.g. *dnaE*, Figure 2D), as well as those that encode gyrase (*gyrA*, *gyrB*), primase (*dnaG*), ligase (*ligA*), and the single-stranded binding protein (*ssb*) (Table S3). *polA*, encoding DNA polymerase I, was the key exception, as it showed weak diel periodicity with a night-time maximum (Figure 2D). The weak periodicity may reflect *polA*'s additional role in DNA repair [37], as DNA photodamage during the daytime is likely to be a significant challenge to this high-light adapted strain.

This direct comparison of the timing of cell division and DNA synthesis with the transcriptome reveals a rather striking choreography of cell cycle progression in *Prochlorococcus*. With few exceptions, the expression of cell cycle-related genes is periodic in a way that suggests a “just-in-time” transcription of genes encoding key steps in the cells progression through the cycle. We do not know if this results in a “just-in-time” translation of the mRNAs into protein, and if so whether such a boost in protein abundance could play a role in triggering these cell cycle events. None the less, the close match between the periodicity of the genes responsible for cell cycle progression and progression itself is striking.

### Photosynthesis

**Figure 3:**
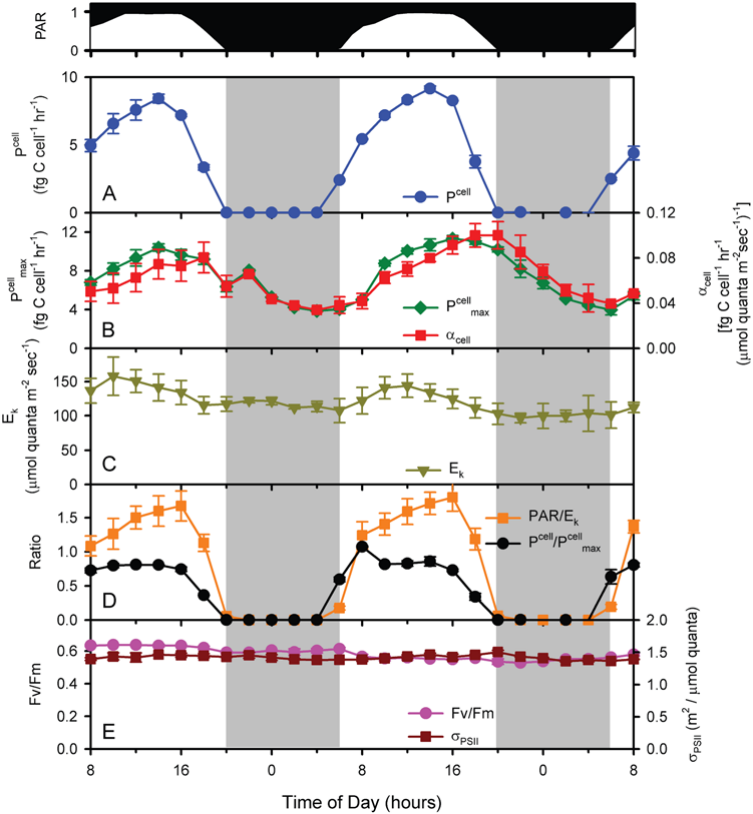
Photosynthesis parameters measured over the time course of the experiment. (A) P^cell^ – cell-normalized photosynthetic rate; (B) P^cell^_max_ – maximum light-saturated photosynthesis, α^cell^_max_ – maximal instantaneous light utilization; (C) E_k_ – maximum light intensity that can be used by the cells; (D) P^cell^/P^cell^_max_, PAR/E_k_; and (E) Fv/Fm and σ_PSII_ are shown (see text for details).

As one would expect, cell-normalized photosynthetic rate (P^cell^, ) directly followed the diel light cycle with peak rates of 8.8±0.5 fg C cell^−1^ hr^−1^ occurring at mid-day (Figure 3A). Integrated photosynthesis over the 24 hour period averaged 82.5±0.5 fg C cell^−1^ d^−1^ for the two days, which represents the daily gross photosynthesis per cell. *Prochlorococcus* has an average cellular carbon content of ∼53 fg cell^−1^ [38], thus this would be the net carbon fixation needed in a day for a cell to double. Since these cultures are doubling once per day, one can conclude from this that the cell respires and/or excretes roughly a third of the carbon it fixes through photosynthesis.

Also as expected, photophysiological parameters were not static over the light dark cycle. For example, both P^cell^_max_ (maximum light-saturated photosynthesis – a measure of photosynthetic capacity) and α^cell^_max_ (maximal instantaneous light utilization – a measure of photosynthetic efficiency, see Materials and Methods) had strong periodicities (Figure 3B), with the former reaching a maximum at mid-day, and the latter reaching one closer to dusk. Minima for the two occurred right before dawn. Because P^cell^_max_ and α^cell^_max_ were not in exact phase and did not have the same changes in amplitude, the light saturation index [39], E_k_ (P^cell^_max_/α^cell^_max_, which is a measure of the maximum light intensity that can be used by the cells) also oscillated with the diel cycle (Figure 3C). It is particularly noteworthy that E_k_ was highest when photons were most abundant (Figure 3C), indicating that the photosynthetic machinery of the cell is running near its maximal capacity (i.e. P^cell^/P^cell^_max_≈1) for a large portion of the day (Figure 3D), even though optimal light utilization efficiency (α^cell^_max_) may not be achieved. Achieving this maximal energy throughput throughout the day comes at the cost of not using all available photons (i.e. PAR>E_k_) for most of the day (Figure 3D), even though this excess light energy does not cause photodamage as evidenced by the invariant Fv/Fm and σ_PSII_ (Figure 3E). Overall, this may be an effective strategy to minimize excess photosynthetic capacity, and the respiratory costs associated with it, thus realizing the highest overall photosynthesis/respiration ratio even though there is additional energy available that could be used. In addition, other sinks for photosynthetic reducing power beyond carbon reduction likely represent important pathways [40]. Thus the diel variability in these photosynthesis parameters demonstrates that although light availability is the proximal factor regulating photosynthetic rates, the photophysiology of MED4 is continually acclimating over the diel cycle and/or cell cycle to maintain balance between light availability and efficiency of utilization.

**Figure 4:**
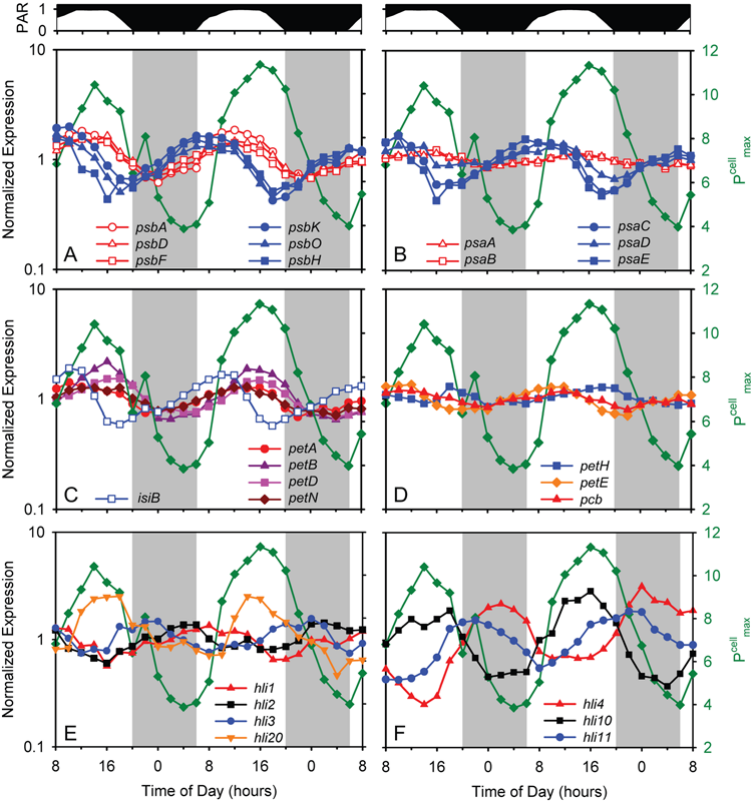
Expression time course of photosynthesis genes. (A) Photosystem II genes *psbA*, *psbD*, *psbF*, *psbH*, *psbK*, and *psbO*; (B) photosystem I genes *psaA*, *psaB*, *psaD*, *psaE*, and *psaC*; (C) Photosynthetic electron transport chain genes *petA*, *petN*, *petB*, *petD*, and *isiB*; and (D) low-periodicity photosynthesis genes *pcb*, *petE*, and *fnr*; and representative high light inducible protein (HLIP)- encoding genes of the (E) single copy - *hli1*, *hli2*, *hli3*, and *hli20* - and (F) multiple copy – *hli4*, *hli10*, and *hli11* - class, with peak abundances at different times over the photocycle are shown. For comparison, P^cell^_max_ (see Figure 3B) is also reported (green). For clarity, error bars representing sample-to-sample variability in gene expression are not shown.

Given this finely tuned physiology, it is not surprising that the expression of many of the underlying genes had strong periodicity in other cyanobacteria [41]–[44] as well as in MED4 (this study). Periodicity patterns of photosynthesis genes fell into 4 clusters (Table 1). Expression of approximately half of photosystem (PS) II genes, including reaction center genes *psbA* and *psbD* (encoding D1 and D2 respectively), as well as *psbC* (CP43) and *psbF*, co-varied with light intensity, with maxima at mid-day, and minima in the middle of the night (Figure 4A, Table S4), consistent with patterns observed by Garczarek et al (2001) and Holtzendorff et al. (2008) in their diel study of selected genes in *Prochlorococcus* PCC 9511. F_v_/F_m_, a measure of the efficiency of PSII, did not change over the course of the experiment (Figure 3E) indicating that the differential expression of photosystem II genes and subsequent protein turnover and reaction center repair was able to mitigate against the damage to PSII [45]–[47]. This is further supported by only minor (<10%) diel changes in the PSII cross-section (σ_PSII_) (Figure 3E). Together, these observations suggest that MED4 may maintain PSII reaction center integrity through changes in gene expression.

A second group of PSII genes including components of the reaction center (*psbK*, *psbO* and *psbH*) peaked earlier in the day — mid-morning — during the G1 phase (Figure 4A, Table S4), and is likely tied to the *de novo* synthesis of reaction centers after cell division [48]. PSI genes largely peak in expression at the same time, except for *psaA* and *psaB* (both PSI core proteins) which display a very low amplitude of expression, with a peak in mid-afternoon (Figure 4B). These results might lead one to hypothesize that the reaction center core proteins of both PSII and PSI, as well as about half of the proteins associated with PSII, are responding directly to light intensity [49], [50] while the remaining PSII and PSI genes are more closely tied to cell cycle processes (i.e. biomass production beginning at sunrise).

Other genes encoding proteins involved in photosynthesis also displayed periodic expression. For example, genes associated with the photosynthetic electron transport chain (PETC) including *isiB* (encoding flavodoxin), and *petA*, *petB*, *petD*, and *petN* (encoding subunits of the cytochrome b_6_f complex) have maxima just prior to or during the period of maximum light intensity (Figure 4C). This suggests that either the components of the PETC are becoming damaged because of oxidative stresses, such as with PSII, or that MED4 is up-regulating the throughput capacity of PETC in response to elevated excitation pressure. The timing of the maximum in maximum photosynthetic capacity (P^cell^_max_) is coincident with the expression maximum of many PETC genes suggesting that PETC throughput increases shortly after noon. It has been shown in the field and laboratory, via changes in the turnover time of PSII (1/τ_PSII_), that phytoplankton can quickly regulate PETC throughput as a mechanism to maintain a maximal P^cell^_max_ in spite of damage to upstream processes (such as the PSII core) [51]. For unknown reasons, other PETC genes did not exhibit strong diel periodicity, including genes encoding plastocyanin (*petE*) and ferredoxin NADP oxidoreductase (*petH*), and the chlorophyll-binding light harvesting complex protein (*pcb*) (Figure 4D).

In general, the diel variation in these photophysiological parameters and the expression of selected genes was consistent with those observed by others for *Prochlorococcus* (PCC 9511) [5], [52], but Bruyant et al. (2005) found that the photochemical efficiency of PSII (F_v_ /F_m_) and the absorptional cross section of PSII (σ_PSII_) varied inversely with light level, while we found little difference in F_v_/F_m_ over the diel cycle. They also observed stronger diel variation in the antenna protein Pcb gene transcript [52] than we did (Figure 4D and Figure S3). We speculate that these differences may be related to the 4-fold lower photon flux used in our study (232 µmol quanta m^−2^ s^−1^ maximum) relative to theirs (912 µmol quanta m^−2^ s^−1^ maximum), perhaps resulting in less stress on the photosystems. Differences in strains used (MED4 versus PCC 9511) may also have played a role.

### High-light inducible proteins (HLIPs)

High-light inducible genes encode a family of photosystem associated proteins in cyanobacteria [53], [54] that are upregulated in response to environmental perturbations such as nutrient, light and temperature stress [29], [30], [55], [56] and provide a fitness advantage during exposure to high light [55]. They are thought to be involved in the protection of the photosystems from excess light energy although the mechanism for this is under debate [53], [54], [57], [58]. High-light adapted *Prochlorococcus* ecotypes, such as MED4, have over 20 copies of the *hli* genes [3], [31], [59]. Four of the MED4 *hli* genes are found in almost all marine cyanobacteria in a single copy and their genome context is conserved. In contrast, many of the other *hli* gene types are found in multiple copies in the MED4 genome, are located in genomic islands [31] and are thought to have originated from phages [31], [60]. This made us wonder if these two classes of *hli* genes had distinguishable expression patterns under these optimal growth conditions.

All *hli* genes of MED4 were expressed during our experiment, and most of them were periodic (19 out of 22) (Figure 4E, Table S5). Intriguingly, the 4 single copy *hli* genes that are found in all *Prochlorococcus* (i.e. are “core” genes) each have peak expression at a different time of day, spread over the diel cycle (Figure 4E). One of them (*hli1*) has the same expression pattern (Figure 4E) as *psbH* (Figure 4A), which encodes the PSII gene product to which an HLIP binds in *Synechocystis* PCC6803 [53]. The multi-copy *hli* genes also show peak expression at different times of the day (Figure 4F), but not in any way that distinguishes them from the single copy genes. Expression of both single copy (*hli20*) and multi-copy (*hli10*) genes co-varied with P^cell^_max_ (Figure 4E, F) whereas expression of other *hli* genes however showed no such correlation, peaking at sunrise (*hli1*), sunset (*hli3* and *hli11*), and many even at night (e.g. *hli2* and *hli4*) (Figure 4E, F).

It is striking that there is always at least one *hli* gene upregulated during any four-hour window of the light-dark photoperiod (Figure 4E, F Table S5), suggesting that the different gene products function at discrete stages in the light-dark cycle. They may, for example, serve to keep photosynthetic machinery running near its maximal capacity (i.e. P^cell^/P^cell^_max_≈1) for a larger portion of the day (Figure 3D). Roles *hli* genes play at night are unknown, but their distributed timing of expression suggests that their activities are more diverse than originally thought. Furthermore, fifteen of the multi-copy *hli* genes that displayed diel variation in expression, but none of the single copy *hli* genes, are also upregulated when MED4 is subjected to environmental stressors [27], [29], [30]. Thus it appears, for the multi-copy *hli* genes at least, that they play a role in both life of the cell under optimal growth conditions as well as in response to specific environmental stressors, suggesting multiple levels of regulation.

### Carbon metabolism and aerobic respiration

Transcripts of genes involved in carbon fixation and storage, carbon catabolism, and respiratory electron transport all show marked diel oscillations, and their timing relative to each other, and the light-dark cycle, offers evidence of tight coordination and phasing of these metabolic pathways. Several reactions are used by multiple pathways that are temporally distinct, presenting a regulatory challenge to the cell. Our probing this phenomenon identified regulatory genes that may be important in orchestrating the flow of carbon and energy in the cell over the course of the light-dark cycle.

#### Carbon fixation and storage

**Figure 5:**
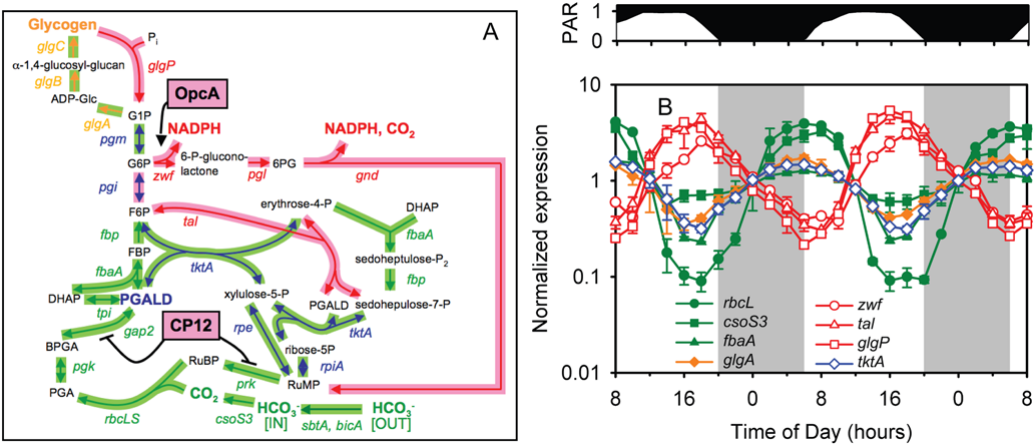
Expression patterns of carbon metabolism genes. (A) Overlay of the Calvin cycle, pentose phosphate pathway, and glycogen metabolism pathways of MED4. Genes and enzymatic reactions (arrows) are color coded by function: green for Calvin cycle, orange for glycogen biosynthesis, red for glycogen catabolism and pentose phosphate pathway, and blue for genes and reactions that are shared between Calvin cycle or glycogen biosynthesis and pentose phosphate pathway. Reactions activated (arrow) or inactivated (dash) by OpcA and CP12 are noted. Peak gene expression was either at sunrise (light green shading of arrows) or sunset (pink shading of arrows and boxes). (B) Expression time course of selected Calvin cycle (green), glycogen biosynthesis (orange), pentose phosphate (red), and dual function (blue) genes. Selected genes are *rbcL*, *csoS3*, *fbaA*, *glgA*, *zwf*, *tal*, *glgP*, and *tktA*. Error bars represent one standard deviation of the mean for the replicate cultures.

The entire suite of genes encoding the pathway for carbon fixation and glycogen biosynthesis in MED4 [3] was maximally expressed at dawn (Figure 5 A,B, Table S6). This is consistent with studies of selected genes in this pathway in *Prochlorococcus* [5], [8], and is the molecular mechanism initiating the conversion of CO_2_ to biomass observed in our physiological analyses (P^cell^, Figure 3A). Bicarbonate (the predominant source of inorganic carbon in the oceans) is first converted, by carbonic anhydrase (*csoS3*), to carbon dioxide in the carboxysome [61]. This is fixed by Rubisco (*rbcLS*) and proceeds through the Calvin cycle, generating net phosphoglyceraldehyde (PGALD) for anabolism. Some of the fixed carbon is also diverted to biosynthesis of the carbon and energy storage molecule glycogen (via *glgA*, *glgB*, and *glgC*). The anticipatory up-regulation of genes during the dark period is likely responsible for the immediate increase in photosynthetic activity (P^cell^, Figure 3A) and biomass (Figure 2A) observed once the light energy could be captured.

#### Carbon catabolism

Energy for the nighttime activities of *Prochlorococcus* cells (e.g. cell division, nucleotide biosynthesis) likely comes in the form of NADPH, which is generated from the catabolism of stored glycogen via the oxidative pentose phosphate pathway [3] (Figure 5A). Genes for glycogen degradation (e.g. glycogen phosphorylase, *glgP*) and the oxidative pentose phosphate pathway in MED4 had peak expression at sunset (Figure 5A,B, Table S6), thus apparently maximizing their potential for nighttime use of their products, as has been noted for other cyanobacteria [41], [43], [44]. Notably, the pentose phosphate pathway shares several reactions with the Calvin cycle. As discussed below, *Prochlorococcus* appears to use several mechanisms to regulate these two intersecting pathways.

#### Respiratory electron transport

In cyanobacteria, respiration occurs in both the cytoplasmic and thylakoid membrane, and in the latter, shares much of the electron-transport machinery with photosynthesis. A key component in both respiration and cyclic photosynthesis is NAD(P)H dehydrogenase. *Prochlorococcus* has two NAD(P)H dehydrogenases, one of the canonical type I (NDH-I) and one of type II (NDH-II) [3]. The latter, composed of a single protein subunit thought to play a regulatory role in other cyanobacteria [62], was not expressed at detectable levels in our study (Table S4). The former are multiprotein complexes, consisting of at least 15 subunits in *Synechocystis* PCC 6803 [62]. MED4 has homologs to all 15 subunits, including 2 paralogs of *ndhD*: PMM0150 and PMM0594. In freshwater cyanobacteria, several complexes of NDH-I exist that contain different paralogs of NdhD and NdhF and that have distinct functions: respiration, cyclic electron transport of photosystem I, and carbon dioxide uptake (the latter is absent in *Prochlorococcus*) [63]–[65]. Like most of the other *ndh* genes, *ndhD* paralog PMM0150 peaked at sunset, consistent with a role in respiration (Table S4). In sharp contrast, *ndhD* paralog PMM0594 peaked at sunrise (Table S4). These results suggest that MED4 has two different NDH-I complexes: one containing the NdhD encoded by PMM0594 that functions in cyclic electron transport of PSI during the day, and a second one containing the NdhD encoded by PMM0150 for aerobic respiration at night.

**Figure 6:**
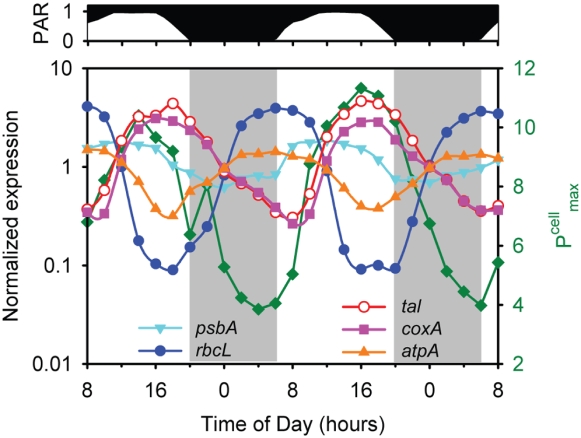
Relationships of carbon and energy metabolism. Expression time course of representative genes involved in photosynthetic electron transport (*psbA*), Calvin cycle (*rbcL*), pentose phosphate pathway (*tal*), respiratory electron transport (*coxA*), and the proton-translocating ATPase (*atpA*) is shown. For comparison, P^cell^_max_ (see Figure 4B) is also reported (green squares). For clarity, error bars representing sample-to-sample variability in gene expression are not shown.

In both photosynthesis and respiration, electrons are passed to the plastoquinone pool, then to cytochrome *b_6_f* [41], plastocyanin, and then photosystem I (photosynthesis) or cytochrome *c* oxidase (respiration). Given that cytochrome *b_6_f* is used by both photosynthesis and respiration, it is unclear which process would be favored in a periodic expression pattern. Genes encoding *b_6_f* peaked at mid-day in our experiment (Figure 4C and Table S4), suggesting that the product of this gene is in greater demand for photosynthesis than respiration. We postulate below a similar explanation for other dual-use enzymes that are found in the Calvin cycle and the pentose phosphate pathway. Conversely, for cytochrome *c* oxidase, which is used only in respiration, all subunits had maximal expression at sunset and minimal expression at sunrise (Figure 6 and Table S4). This pattern matches that of glycogen degradation and the pentose phosphate pathway, which presumably supplies NADPH and its electrons for respiration. It remains to be determined what fraction of NADPH from the pentose phosphate pathway is used for respiration and what fraction is used for other processes, such as nucleotide reduction and combating oxidative stress.

ATPase couples the proton gradient, created by electron transport, to ATP synthesis. ATPase should function to generate ATP during both photosynthesis and respiration. It is thus curious that the diel expression pattern of all subunits match those of the Calvin cycle genes (Figure 6, Table S4). This invites the hypothesis that gene expression has been optimized to handle the greatest *demand* for ATP (carbon fixation), rather than the potential greatest *production* of ATP (photosynthetic light reaction and/or respiration).

#### Dual-use enzymes and intersecting pathways

Metabolic networks often employ the same enzyme, and the chemical transformation it catalyzes, for several different purposes. Indeed this is the case in cyanobacteria, particularly vis a vis pathways that are partitioned between night and day. While this could provide efficiency, it presents a problem: If an enzyme is used at day and at night, and expression of its gene is periodic (as are most genes in *Prochlorococcus*), should it be maximally expressed at day or at night?

One of the most striking examples of dual-use enzymes in cyanobacteria is the shared enzymes of the Calvin cycle and the pentose phosphate pathway. Six reactions, catalyzed by five enzymes, are shared between these pathways (Figure 5A, blue arrows). Like most genes in *Prochlorococcus*, the transcripts of these exhibit a diel periodicity: all with peak expression at sunrise and minimal expression at sunset, similar to the expression of the Calvin cycle genes. Assuming a tight coupling between the timing of gene expression and protein levels, this may seem counterproductive for nighttime respiration. It is possible, however, that flux through these reactions is more intense when the Calvin cycle is operating, so greater quantities of these enzymes (and hence transcripts) are needed during the day. Additionally, we note that four of the six shared daytime-maximal reactions run in opposite directions in the two pathways. Intriguingly, work in other organisms has shown that the equilibrium constants for these four reactions all favor the pentose phosphate pathway direction: ribulose-5-P isomerase (*rpiA*), ribulose-5-P epimerase (*rpe*), and both transketolase (*tktA*) reactions [66]. Given these equilibria, particularly that of the final transketolase reaction (*K*_eq_ = 17), it is plausible that smaller quantities of enzymes at night are sufficient to yield significant flux through the pentose phosphate pathway, and that the diel periodicity of the transcripts that encode these enzymes serves to keep these channels equally open for both the Calvin cycle and the pentose phosphate pathway.

The alternation in carbon flow between these two pathways raises another issue. How does the cell direct carbon in the required direction and mediate the oscillation between these two pathways? Perhaps translational control is sufficient; abundance of enzymes exclusive to one pathway could steer the overall flux in the required direction. It appears, however, that additional regulatory mechanisms, involving the post-translational regulatory proteins, CP12 and OpcA, are operating to control the switch between Calvin cycle and pentose phosphate pathway. CP12, an intrinsically unstructured protein, has been shown to directly inhibit the Calvin cycle during nighttime conditions in both cyanobacteria and green plants [67], [68]. In our data set, the gene encoding CP12 (PMM0220) is maximally expressed in the evening (Figure 5A). The oxidizing conditions of a cyanobacterial cell at night are known to trigger CP12 to bind and deactivate the Calvin cycle enzymes phosphoribulokinase (PRK, *prk*), and glyceraldehyde-3-P dehydrogenase (GAPDH, *gap2*) [67]. Thus, nighttime expression of CP12 likely serves to shut off key steps in the Calvin cycle of MED4 to let the pentose phosphate pathway proceed unhindered. At the same time that the gene encoding CP12 is induced, so is that for OpcA (Figure 5A), an allosteric effector of the first enzyme of the oxidative pentose phosphate pathway, glucose-6-P dehydrogenase (G6PDH, *zwf*). OpcA is known to increase G6PDH affinity for glucose-6-P more than 100-fold in other cyanobacteria [69]. From these expression patterns, we suggest that at the same time CP12 restricts carbon flow through the Calvin cycle, OpcA appears to redirect carbon flow through the pentose phosphate pathway. Together with the alternate phasing of expression of the genes encoding the pathway enzymes, induction of regulatory proteins that activate or deactivate key enzymes of the two pathways may be the crucial events that facilitate the temporal separation of the Calvin cycle and pentose phosphate pathway.

#### Offset of transcripts for photosynthetic light and dark reactions

We found an interesting difference in phasing between expression of the Calvin cycle genes and those that encode the light reaction of photosynthesis, which provide the Calvin cycle with energy and reducing power (Figure 6). Most of the genes of the photosynthetic electron transport chain reach peak expression levels in the middle of the light period, where as Calvin cycle genes, such as *rbcL*, had peak expression levels at dawn and transcript levels were minimal toward the end of the day. This may account for the slight uncoupling of P^cell^_max_ and α^cell^_max_ (Figure 3B): maximal carbon fixation via the Calvin cycle may occur before maximal light utilization due to the offset in timing of the synthesis of the proteins involved (Figure 6).

Assuming our inference from transcript levels is correct, why does expression of Calvin cycle genes precede expression of the light reaction genes? Perhaps it allows the cell to take immediate advantage of reducing power at sunrise, thus minimizing the dependency of photosystem usage during periods of high (and damaging) light intensity later in the day. Additionally, significant down-regulation of *rbcLS* in the afternoon may help limit the amount of photorespiration (i.e. oxygenation of ribulose-1,5-bisphosphate, rather than carboxylation) during periods of high light and O_2_ production. All of these interpretations remain hypotheses until they can be explored at the protein and metabolome levels rather than transcript level alone.

In summary, over the course of the photocycle, the energy source for *Prochlorococcus* undergoes dramatic variation. The amount of light available at a particular time determines the source of electrons that are used for NADPH production— either water or glycogen. Our transcriptome analysis has generated hypotheses about how the transitions to the different modes of energy and carbon metabolism are mediated at the level of gene expression. Genes of the oxidative pentose phosphate pathway and the respiratory electron transport chain, which together turn glycogen into NADPH and then ATP, cycle with sunset maxima and sunrise minima — 180° out of phase with those of the Calvin cycle (Figure 5B, Figure 6). These patterns, plus those of the regulatory proteins CP12 and OpcA (see above), suggest how the *Prochlorococcus* cell transitions from daytime photosynthetic carbon fixation to the nighttime shutdown of the Calvin cycle and induction of the respiratory pathway, which likely accounts for the observed nighttime decline in photosynthetic capacity (P^cell^_max_) (Figure 3B).

### Diel periodicity of nutrient acquisition and assimilation

**Figure 7:**
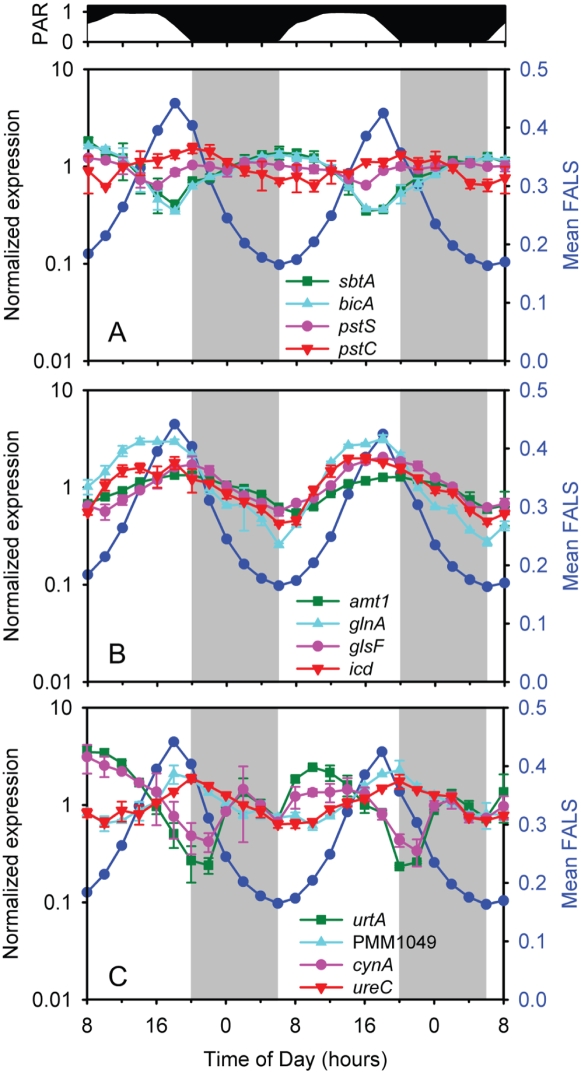
Expression time course of nutrient transport and assimilation genes. (A) Transporter genes for bicarbonate (*sbtA*, *bicA)* and phosphate (*pstS*, *pstC*); (B) ammonium transport (*amt1*) and assimilation (*glnA*, *glsF*, *icd*); and (c) transporter genes for urea (*urtA*), oligopeptides (PMM1049), cyanate (*cynA*), and for a urease subunit (*ureC*) are shown. Cell size (FALS) variation over the photocycle is also presented (dark blue circles) for comparison. Error bars represent one standard deviation of the mean for the replicate cultures.

Nutrient transporters are a critical link between the cell and its environment. One might predict *a priori* that the transporters for carbon, phosphorus, and nitrogen are maximally expressed near the time of greatest demand by the cell each element. In the mildly-alkaline oceans, the vast majority of inorganic carbon is in the form of bicarbonate, and we might expect demand to be highest during the day, when cellular biomass increases (Figure 2A). MED4 contains homologs to two sodium-bicarbonate symporters, *sbtA* and *bicA* [63], that are likely in the same operon. As predicted from supply and demand considerations, expression of both genes cycled synchronously with the Calvin cycle and carboxysome genes (Figures 5A, 7A), sharing the same cluster (16) with most of them (Table S6).

P-limitation exerts a strong selective force on the composition of *Prochlorococcus* and its genome [28], as evidenced by the fact that most of cellular P is in DNA and RNA [70], and essentially none in phospholipids [71]. We expect the cell's greatest demand for P to be during the day-to-night transition, i.e. the period of DNA replication (Figure 2D), high total mRNA accumulation (Figure 1), and peak expression of the nucleotide biosynthesis genes (Table S1). With the greatest demand for P during the evening, the expectation was that peak expression of phosphate uptake genes would also be in the evening. Indeed, this was the case for the trans-membrane and ATP-binding cassette components of the ABC-type phosphate transporter (together the *pstCAB* operon)(Figure 7A). In contrast, *pstS*, which encodes the periplasmic phosphate binding protein component of the transporter has high transcript levels with very weak periodicity with a late-night maximum (Figure 7A). We speculate that this near-aperiodic expression serves to maintain a constant (high) concentration of PstS in the periplasm, and trap any phosphate that may enter throughout the photoperiod, while transport *per se* is maximized at night, at the time of highest demand.

Previous studies have shown that alkaline phosphatase (encoded by *phoA*), which cleaves phosphate from organic sources in the periplasm, and an alkaline phosphatase-like protein of unknown function (encoded by *dedA*) exhibit contrasting expression patterns in MED4: *phoA* is highly upregulated under P-starvation while *dedA* is not [28], [72]. In this study we observed measurable *dedA* expression with a maximum in the mid-afternoon, while *phoA* displayed periodicity similar to that of phosphate uptake genes, with peak expression just after dark (Table S7), just anticipating the greatest cellular demand for P. We postulate that *dedA* may be responsible for the low constitutive alkaline phosphatase activity (APA) that has been documented for MED4, and other *Prochlorococcus* strains that lack *phoA* [72]. Measuring APA over a diel cycle under both replete and P-starvation conditions will help tease apart the roles of these and other phosphatases.

The cellular demand for nitrogen is more complex than that for phosphorus, with the majority of nitrogen contained in proteins in addition to nucleic acids. Nitrogen demand for protein synthesis is likely to closely follow that for mRNA which shows a bimodal pattern of expression at both sunrise and sunset (Figure 1), but is also distributed at moderate levels throughout the day. Given this complexity, it is difficult to postulate any sort of supply/demand relationship for this element without proteomic data. Thus we simply offer some selected observations regarding the periodicity of expression, or lack thereof, of N-related genes.

Amino acid synthesis genes show bimodal patterns of expression (Table S1) with peaks at sunrise and sunset, while expression of ribosomal (protein) genes is highest throughout the night. We note further that the expression of different genes in the synthesis pathway for the same amino acid peak at different times over the cycle suggesting complex patterns in N-demand for protein synthesis. Transcription of the ammonium transport gene (*amt1*) peaked in the evening, near sunset (Figure 7B). Transcript levels of *amt1* were at least an order of magnitude higher than for other nitrogen metabolism related genes (data not shown), and displayed low cyclic amplitude over the diel cycle (Figure 7B). This suggests that, similar to the phosphate periplasmic binding protein encoded by *pstS*, there is a need for constant high expression of Amt1 to ensure efficient scavenging of any available ammonium from the nutrient deplete waters *Prochlorococcus* inhabits in nature. The ammonium assimilation pathway genes exhibited the same periodicity as the transporter (Figure 7B), peaking in the evening. Ammonium is assimilated into organic compounds via the glutamine synthetase (GS) – glutamate synthase (GOGAT) pathway (encoded by *glnA* and *glsF* respectively) and the carbon skeleton for its incorporation is 2-oxoglutarate (2-OG) [73]. The ammonium assimilation pathway genes exhibited the same periodicity as the transporter (Figure 7B), peaking in the evening. 2-OG is produced from isocitrate by isocitrate dehydrogenase (*icd*) which was also maximally transcribed in the evening (Figure 7B). This suggests that the major source of the carbon skeleton (2-OG) for ammonium assimilation is not phosphoglyceraldehyde generated directly from photosynthesis, but rather from glycogen stores.

Although ammonium is preferred [73], *Prochlorococcus* can utilize different sources of nitrogen for growth. Urea and cyanate can serve as nitrogen sources in *Prochlorococcus* [30], [74]. Although they were not in the media during this experiment, their transporters (*urtAB* and *cynA*) were expressed with a complex pattern of transcription with maxima soon after sunrise and a secondary peak at night (Figure 7C). Both of these genes encode ABC-type transporters and their peak expression coincides with that of the ATPase gene (Figure 6). Urease genes (which convert urea to ammonium) had maximal expression in the evening (Table S8), consistent with the timing of expression of ammonium transport and assimilation genes but different to that for the urea transporter genes. Finally, recent data suggests that *Prochlorococcus* can take up methionine and leucine, and that their accumulation is significantly higher at dusk than at dawn [75]. This matches the timing of expression of ammonia uptake and assimilation genes, as well as that for the predicted oligopeptide permease gene (PMM1049) (Figure 7C) in our experiment.

Previously reported P and N starvation responses in *Prochlorococcus* MED4 revealed the up-regulation of many genes besides those directly involved in uptake and assimilation [28], [30]. We examined the periodicity of these genes in our experiment and found that their behavior fell into two distinct subsets: some had transcription patterns similar to phosphorus and nitrogen assimilation genes (data not shown), and some were not expressed above background at any time point. Genes in the first group therefore appear to be subjected to multiple layers of regulation. Their induction during nutrient starvation indicates a role in stress response while their diel oscillation suggests that they also play integral roles in nutrient assimilation even in cells grown under optimal conditions. Genes in the latter group, however, may be stress-response specific being highly induced from background levels during nutrient starvation. These include PMM1403 and PMM0721, genes of unknown function which are upregulated during P-starvation [28], and the nitrogen transcriptional activator *ntcA* and PMM0958 a gene of unknown function which is the most highly upregulated gene during N-deprivation [30].

### Regulation

The mechanisms that regulate and choreograph the cyclic gene expression patterns we have described are yet to be unveiled. It is likely, however, that they involve (1) transmission of light as a signal for gene expression through a photoreceptor-regulatory pathway and (2) a diel oscillator of some sort. While the expression of some genes, such as *psbA*, varies in direct proportion to available light, this is not true for the majority of periodic genes. In particular, if light is the sole trigger for up or down regulation, it is difficult to reconcile this with the night-time induction of genes such as *rbcL* that seem to anticipate the coming of dawn. Hence the most likely “master controller” of the transcriptome would appear to be some sort of endogenous oscillator like a circadian clock. Yet as discussed above, *Prochlorococcus* lacks key components of the cyanobacterial clock, such as *kaiA* and *cikA* and does not display cyclic gene expression under constant conditions [12]. Are there any clues as to regulation in the patterns of expression of the known clock genes in *Prochlorococcus*?

**Figure 8:**
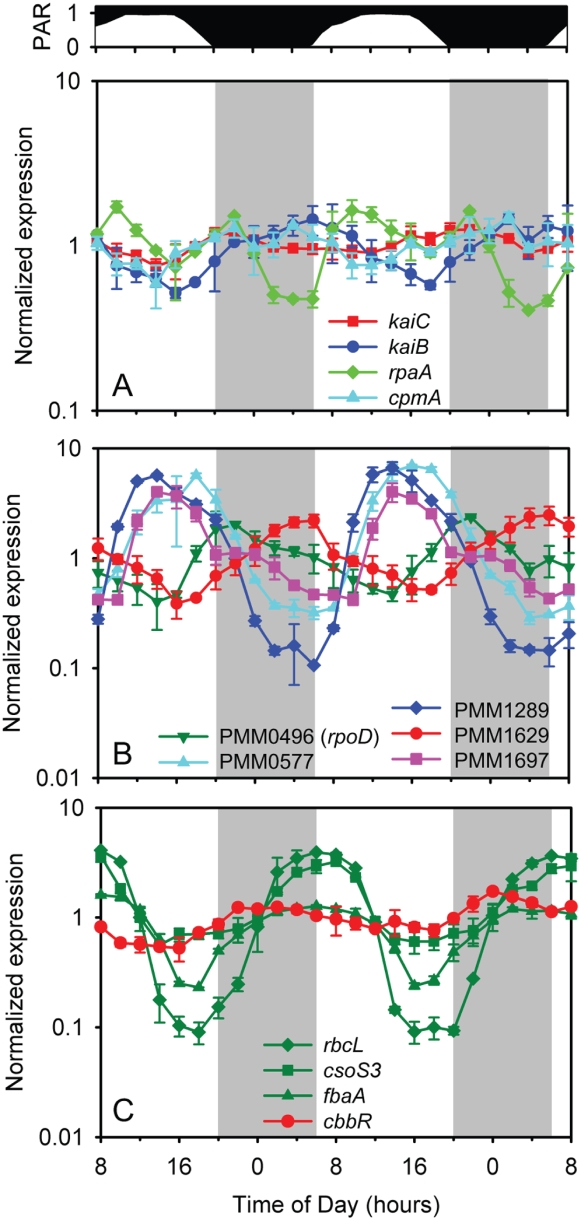
Expression time course of regulatory proteins. (A) Circadian clock genes *kaiB* and *kaiC*, and clock output genes *rpaA* and *cpmA*; (B) Sigma factor genes PMM0496 (*rpoD*), PMM0577, PMM1289, PMM1629, and PMM1697; and (C) *cbbR*, and representative Calvin cycle genes *rbcL*, *csoS3*, and *fbaA* are shown. Error bars represent one standard deviation of the mean for the replicate cultures.

The *kaiB* clock gene of MED4 exhibited strong diel periodicity in our experiment, with a maximum at dawn and a minimum at sunset (Figure 8A). *kaiC* showed low, albeit significant diel periodicity, peaking near the onset of darkness. While the *kaiB* pattern resembles that reported by Holtzendorff et al. (2008) for *Prochlorococcus* PCC 9511, the *kaiC* pattern is just the opposite: they found that *kaiC* peaked at dawn, in phase with *kaiB* (albeit with a small secondary peak just after dark, in phase with the peak we observed). This difference is puzzling. At this time, we can only add that we found the same weak periodicity of *kaiC*— with a small peak after the onset of darkness — in our Pilot Study (see methods) using quantitative reverse transcription PCR (Figure S3), as was observed in our study with the arrays.

MED4 has homologs to *sasA* and *rpaA*, which in another cyanobacterium encode the histidine kinase and cognate response regulator that are essential for transmission of the clock's output to the genome [23], [76]. Transcripts of the *sasA* homolog oscillate in a pattern almost identical to *kaiC* in our experiment (Table S9), while the *rpaA* homolog peaks twice per 24-hour period, at 10:00 and 12 hours later at 22:00 (Figure 8A). *cpmA* is also involved in clock output [77], and the homolog of this gene in MED4 shows weak but significant periodicity, peaking just prior to *kaiB* at night (Figure 8A). Hence, all of the homologs of circadian clock-related genes that MED4 possesses exhibit diel periodicity in transcript abundance. The significance of these periodicities is currently unknown, given that SasA protein accumulation in *Synechcoccus elongatus* PCC 7942 was constitutive over a light-dark photocycle [23], and that two-component regulatory systems such as SasA-RpaA are themselves regulated primarily at the post-translational (phosphorylation state) level.

All five sigma factors in MED4 (one group I plus four group II) cycle with unique phase relations to the diel photocycle (Figure 8B). Holtzendorff el al. (2008) reported the different periodicities of two of the sigma factors (PMM1629 and PMM1697), and here we confirm and extend those results to include all five. PMM1289, PMM0577, and PMM1697, cycle similarly but not identically: PMM1289 begins to accumulate in early morning, peaks at mid-day, and is followed by PMM0577 and PMM1697 with a 2 hour offset (Figure 8B). The predicted principal (group I) sigma factor (*rpoD*, PMM0496) peaks two hours after the onset of darkness, and the final sigma factor (PMM1629) peaks at dawn. PMM1629 has the same phasing as *kaiB*, which raises the possibility that the former regulates expression of the latter, although the reverse scenario might also be true. In addition, PMM1629 is also in expression cluster 15, which is significantly enriched with Calvin cycle and ATPase genes (Table 1), raising the possibility that it controls expression of a photosynthesis regulon.

We suspect that differential phasing of expression of the sigma factors may contribute significantly to the diel expression patterns of the rest of the transcriptome. In other cyanobacteria, the inactivation of group II sigma factors can cause defects in *psbAI* and *kaiB* circadian expression [78], [79], and they are thus thought be involved in transducing the clock output signal to the genome: While group II genes are transcribed in phase (in contrast to MED4), they are thought to confer the observed staggered variation in expression of target genes through differential phasing of activity [78]. It is argued that the sigma factors all compete for the core RNA polymerase, but are activated (perhaps via translation) at different times in the photoperiod, thus with their different affinities for both core polymerase and the suite of promoters within the genome, effectively turn different sets of genes on and off at different times [78]. In support of this hypothesis, cross-regulation between the group II sigma factors during the day and during transitions from dark to light has been reported in *Synechocystis* PCC 6803 [80], [81], and one (SigB) regulates transcription primarily in the dark, while the another (SigD) does so primarily in the light [82].

The transcripts of most (46 of 54) of the other putative transcription factors [3] cycle over the light – dark cycle; only five of those expressed were aperiodic, and only four were below the signal threshold to be considered expressed (Figure 8, Table S9). Thus a large fraction of the transcription factors appear to be active during optimal growth under a light-dark cycle. Might the diel periodicities of these regulators establish the periodicities of other genes over the photocycle?

There are two examples that invite investigation in this regard — one involving carbon acquisition and the other nitrogen acquisition. The expression of the Calvin cycle genes is concurrent with, or proceeded by, the increased expression of PMM0147 (Figure 8C). This is a homolog of the regulatory protein gene *cbbR*, paralogs of which are known to play roles in carbon metabolism in *Synechocystis* PCC 6803 [83]. The MED4 gene PMM0147 is most similar to the *lysR* paralog, which is essential in *Synechocystis* PCC 6803, and believed to regulate *rbcLS* in this system. As the MED4 genome lacks any other CbbR-type paralogs, it appears that PMM0147 is a functional ortholog of *lysR*, and its expression pattern further implicates it in the diel regulation of the Calvin cycle genes, including *rbcLS*. Regarding N-acquisition, PipX is a transcriptional co-activator that is required for NtcA-dependent transcription of nitrogen metabolism and transport genes under N-stress [84]. The *pipX* gene in MED4 displayed maximal expression at sunset (Table S9) as did most of the N metabolism genes. It is worth exploring whether the diel periodicity of nitrogen transport and assimilation genes that peak in the evening in *Prochlorococcus* is mediated by the periodicity of *pipX*.

### Comparison of the light-dark entrained transcriptome with that from cells grown in continuous light

Given the tight alignment of the *Prochlorococcus* cell cycle to the light-dark cycle, and the choreographed gene expression and physiology, it is perhaps surprising that *Prochlorococcus* can be maintained at maximal growth rates under continuous-light [85]. To begin to understand the adjustments in cellular physiology that enable growth under continuous light, we compared our diel transcriptional profiles to the continuous light profiles (of nutrient-replete control treatments) from previous experiments with MED4 [28], [30]. If growth in continuous light simply effects the complete desynchronization of the population that has been shown to occur in cultures shifted from light dark cycles to continuous light [12], then we would expect gene expression in continuous light to represent some average expression over all time points of the diel cycle. Instead, we found 39 genes whose RMA-normalized expression is significantly higher (>2-fold, q<0.05) in continuous light compared to the mean over the diel cycle, and 17 genes significantly lower (>2 fold, q<0.05) (Table S10). The products of the former 39 genes include ten high light induced proteins, 3 group II sigma factors (PMM0577, PMM1289, and PMM1629), and five heat shock proteins, including GroEL/GroES. While interpretation of this comparison cannot be conclusive because the integrated 24 hour photon flux in the continuous light experiments was 35–60% that in the diel experiment, and the cells were growing at lower growth rates, it is striking that “stress-related” genes constitute a significant fraction of the overrepresented transcripts under continuous light conditions. A side-by-side comparison of the continuous light and diel transcriptome of cells grown under the same daily integrated photon flux would be instructive, as would a comparison of the transcriptome of cells maintained at the same growth rate, in continuous light and on a light dark cycle. Combined, these experiments would help bring to light the cell's response to continuous illumination at the molecular level.

### General conclusions and future directions

This study brings us one step closer to the broad goal of developing *Prochlorococcus* as a model for integrative systems biology — i.e. to understand its cellular architecture, variability, and the forces that shape the *Prochlorococcus* meta-population in the global oceans. A description of the diel transcriptome of the cell in the context of its photophysiology and cell cycle is essential for the development of metabolic models of the cell. Coupled to future proteomics and metabolomics studies, we will have a more complete understanding of how diel gene expression, and the timing of protein activity, is controlled at the level of transcription, translation, and post-translational regulation. Furthermore, this data set is an invaluable reference for interpreting the growing open-ocean meta-transcriptomics database, in which *Prochlorococcus* transcripts are highly represented [86].

The *Prochlorococcus* system is particularly useful for this type of study because of the tight synchrony of the cells when grown on a light-dark cycle, ensuring that the gene expression patterns reflect what one would measure in an individual cell as it progresses through its cell cycle. This schedule of events appears highly choreographed and aligned with the photocycle. The cell is ‘born’ sometime during the dark period and by the time dawn arrives, the transcripts of the full complement of Calvin cycle and carbon concentrating mechanism genes are maximally abundant, as well as those of many genes encoding members of the photosynthetic electron transport chain. This primes the cell for photosynthesis and net biomass accumulation, which begins as soon as light hits the cell. Expression of some other photosynthetic genes, such as *psbA*, appears to be under a different regulatory regime as they directly track light intensity, peaking at noon. In other cyanobacteria, *psbA* expression is controlled by light (and/or redox state) and the circadian clock [49], [87], [88], and this may be the case for MED4 as well. As dusk approaches, expression of DNA polymerase and other genes involved in DNA synthesis are maximally expressed, closely followed by the onset of chromosome replication (S phase) of the cell. As day transitions to night, genes encoding the divisome become maximally expressed, and the cell undergoes cell division sometime during the night, completing the cycle. The day to night transition is also marked by a switch in energy metabolism from photosynthesis to aerobic respiration, and in carbon metabolism from CO_2_ fixation to catabolism of glycogen, both of which are manifested in the changes in gene expression.

The robust periodicities of gene expression in *Prochlorococcus* suggest strong selection for the coordination of cellular processes in face of the oscillating energy supply. Indeed, relative fitness of *Synechococcus elongatus* PCC 7942 clock mutants has been shown experimentally to be a function of how closely their endogenous period matches that of the environmental light-dark cycle [89]. While we do not have similar direct evidence for *Prochlorococcus*, the temporal partitioning of the expression of Calvin cycle and Pentose Phosphate Pathway genes (Figure 5), for example, suggests that selection under the daily photocycle has shaped these patterns. These two pathways play opposite roles in the cell — the former trades energy for fixed carbon, and the latter does the reverse — yet they share several enzymes. This would pose a significant regulatory challenge for the cell if both were operating at the same time — a challenge that would be exacerbated by the streamlined regulatory system of this cell.

Gene inactivation (which is currently not possible in *Prochlorococcus*), proteomics, and studies that vary the growth rate (see below) should provide valuable tests of the hypotheses about regulation generated by these descriptive data. In this study, the doubling time (approximately 1 day) matched the 24 hour photoperiod. But we know that the length of the DNA synthesis phase (S) is growth rate independent in *Prochlorococcus*, while the pre-and post-synthesis phases expand with generation time [14]. Thus by varying average cell generation time to offset it from the 24-hour photoperiod, one may be able to see which processes are set by photoperiod, and which by growth rate. It would also be informative to study these diel transcription patterns under nutrient limited conditions. One could then ask questions such as: Does oscillation in the availability of a limiting nutrient influence the choreography of the transcriptome in response to the photocycle?

In the oligotrophic ocean, where seasonality is typically weak and conditions generally change slowly, the diel light-dark cycle is one of the principal features governing temporal variation in microbial community function. *Prochlorococcus* is one of the few, if not currently the only, microbe whose transcripts are represented in relatively high abundance in meta-transcriptomics data from the open ocean [86]. Thus this laboratory study of the tempo of expression in *Prochlorococcus* cultures, compared with data from the field, can help inform the design of oceanographic sampling strategies. The strongest contrast in gene expression levels in our study was not, as might have been expected *a priori*, between midday and midnight, but rather between sunrise and sunset. In fact, expression levels of most genes were equivalent mid-day and mid-night, with some on the upswing and some on the downswing. Hence if resources are limited and one cannot resolve the entire light-dark cycle it would be most important to sample and sequence around dawn and dusk to capture the metabolic pulse of a cell like *Prochlorococcus*. As a dominant primary producer in these systems, this pulse may be important in driving that of other organisms in the microbial food web.

Interactions between *Prochlorococcus*, phages that infect them, their protozoan predators, and competing microbes, are all likely influenced by diel cycling, as well as other environmental factors which may in turn influence their responses to the oscillating energy input. While the complexity of the interactions is daunting, we are beginning to develop tools that bring it closer into focus. Novel ocean ecosystem models are under development that begin to embrace the diversity of metabolic possibility among microbes [90], and we are getting closer to cellular systems models of ocean microbes, in part through studies such as this one. Our hope is that in time, these two types of systems biology models will meet in the middle, such that the interactions between the environment and the cell can be explored at multiple levels of organization, from the genome to the ecosystem. This will open new vistas for understanding the nature, evolution, and regulation of microbial processes.

## Materials and Methods

### Pilot Studies

Before executing the comprehensive transcriptome analysis using micro-arrays, a pilot study was conducted on axenic MED4 to determine optimal sampling strategies. Quantitative reverse transcription PCR (QRTPCR) was used to analyze the transcript levels of key genes involved in cell cycle processes (*ftsZ*, *dnaA*), photosynthesis (*psbA*, *pcb*, *rbcL*), the circadian clock (*kaiC*) as well as transcription (*rpoD*). For this study cultures were grown as described below, except on a 12 hour light (approximately 300 µmol Q m^−2^ s^−1^), 12 hour dark cycle (without dawn and dusk). The growth rate of the culture was 0.47 day^−1^. QRTPCR was carried out according to the methods described in [91]. The *rnpB* housekeeping gene was used to normalize RNA between samples. The primers used are shown in Table. S11. Transcript levels of the genes analyzed are shown in Figure S3. We present these results here to show that transcript periodicity patterns of these genes are similar to those determined with the arrays in the actual experiment, even though the culture growth conditions were not identical. For the actual experiment conducted for the arrays, the L∶D cycle was changed to 14:10, with a dusk and dawn simulation (see below), so the cultures would grow at exactly one doubling per day.

### Culture conditions

Axenic strain MED4 was grown in Sargasso Seawater-based Pro99 medium, which provides nitrogen as ammonia and phosphorus as inorganic phosphate. The Pro99 medium was supplemented with 10 mM HEPES buffer (pH 7.5) to maintain pH and prevent CO_2_ limitation [85]. Replicate batch cultures were grown in 10 L volumes within 13.25 L acid-washed glass vessels with slow stirring, at 24±0.2°C. This light level provided maximal growth rate for MED4 under the conditions provided (data not shown). Incubations were performed in a modified Percival Scientific (Boone, IA) I-35LL plant growth chamber. Standard 20 W bulbs and supporting ballasts were replaced with 54 W high-output bulbs and supporting ballasts. Creation of a control device allowed for the voltage-regulated variation in light output from these bulbs. This lighting system was programmed to provide a 14 hour light, 10 hour dark cycle, with a gradual increase or decrease of light at experimental sunrise or sunset, respectively. Sunrise initiated at experimental 06:00, ending at 10:00, and sunset initiated at experimental 16:00, ending at 20:00. Maximum light intensity, at experimental 10:00–14:00 was approximately 232 µmol Q m^−2^ s^−1^.

Every two hours over the 50 hour experiment, 300 mL of the cultures were transferred to centrifuge bottles. Sampling at experimental night time points was performed under very low (<1 µmol Q m^−2^ s^−1^) red light conditions. Cells were pelleted by centrifugation at 10,000 RPM at 20°C, and resuspended in 1 mL RNA resuspension buffer (200 mM sucrose, 10 mM sodium acetate, 5 mM EDTA, pH 5.2) [29], [91]. Samples were snap-frozen in liquid nitrogen and stored at −80°C until processing. At each time point, 3 1 mL aliquots were also prepared for flow cytometry following [92]. To these aliquots, a 0.125% final concentration of TEM grade glutaraldehyde (Tousimis) was added, and after a 10 minute incubation in the dark, these fixed cells were snap frozen and stored in liquid nitrogen.

### RNA isolation and quantification

Total RNA was extracted, purified from DNA, and concentrated following Lindell et al. (2005). For microarray analysis, 2 µg of total RNA was labeled and hybridized to the custom MD4-9313 Affymetrix GeneChips®, following standard protocols [29], [91]. Raw data were normalized by the Robust Multichip Average (RMA) algorithm [93], via the GeneSpring GX 7.3.1 software (Agilent Technologies).

### Flow cytometry and cell cycle analysis

Thawed samples were stained with the DNA stain Hoechst 33342 (0.5 mg ml−1 final concentration) and held at room temperature in the dark for 1 hr prior to analysis following [94], [95]. *Prochlorococcus* were enumerated using a modified EPICS V (Coulter) flow cytometer following [96], [97]. Relative DNA and chlorophyll concentrations were determined using cellular blue and red fluorescence, respectively, normalized to 0.46 µm carboxylate and 0.47 µm YG bead standards (Polysciences), respectively, following [94]. Cell-cycle parameters were determined using FlowJo cell-cycle analysis software v (TreeStar) from DNA histograms and following [98]. No heterotrophic bacteria (i.e. populations without red fluorescence) were detected over the course of the experiment.

### Photophysiology

Photosynthesis irradiance (P-E) curves were measured using the C-14 technique with a conventional photosynthetron [99] as previously described [100], [101]. Briefly, 13 1 ml samples were each inoculated with ∼0.37 MBq H^14^CO_3_, incubated at different light levels in a custom-built, temperature-regulated photosynthetron and terminated after 1 hr with 1N HCl, final concentration. Carbon uptake was quantified using liquid scintillation counting following Barber et al. (1996) [102]. A standard P-E model [103] was optimized to data using a custom written routine following [104] to determine key parameters of photosynthesis, including the light utilization index (α), maximal photosynthesis (P_max_) and light saturation index (E_k_) of the P-E curves as defined by Sakshaug et al. [105]. Rates of photosynthesis for each 2 hr time period in each replicate culture were measured similarly in duplicate except that samples were incubated at ambient light levels with the culture. Single turnover fluorescence induction curves were measured using a Background Irradiance Gradient – Single Turnover fluorometer (BIG-STf) to measure the photosynthetic conversion efficiency (Fv/Fm) and functional absorption cross section (σ_PSII_) of photosystem II (PSII) as a function of background light intensity as previously described [106]. Duplicate samples from duplicate cultures were dark acclimated for >15 mins, after which single turnover fluorescence induction curves were measured over a range of background light levels. Photosynthetic parameters (Fv/Fm and σ_PSII_) were estimated by fitting standard models to data to determine values of *Fo* (initial fluorescence), *Fm* (maximal fluorescence), *Fv* (*Fm*-*Fo*), σ_PSII_ (functional cross-sectional area of PSII) and *p* (PSII connectivity parameter) [107].

### Normalization and computational analysis of Affymetrix arrays

Signal intensities for Affymetrix probe sets were calculated and normalized using the Robust Multi-Array Average (RMA) procedure as implemented in the Bioconductor package *affy* [108]. Additionally, we applied the Microarray Suite (MAS 5.0) and “Golden Spike” normalization schemes to study the influence of the chosen normalization procedure [109]. Although some variation in the calculated signal intensities was observed, the main results of the computational analysis remained unaffected.

The detection of periodic expression was based on Fourier analysis, as a recent comparison showed its superior performance compared to other approaches [110]. After averaging over the corresponding time points in both experimental runs, a Fourier score was calculated for the temporal expression pattern of each gene. The Fourier score is defined as
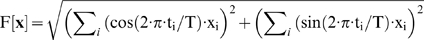
where ***x*** is the standardized expression vector (mean(***x***) = 0; sd(***x***) = 1) for the gene, T is the period (in our case 24 h), and x_i_ is the measured expression at time point *t_i_*.

To assess the significance of the score obtained, the probability of how frequently such a score would be observed by chance has to be calculated. Thus, a background model for the Fourier score *F* was generated by fitting autoregressive processes of the order 1 (AR (1)) to the observed time courses and subsequent calculation of *F* for the generated random expression vectors. Note that the AR(1)-based background models give an improved estimation of the significance of periodic microarray data compared to conventionally used background models based on random permutation [111]. Next, the significance of the measured periodicities was obtained by comparison with the generated background distribution. For each score, a FDR (False Discovery Rate) was calculated representing the fraction of estimated false positives. A FDR-value of 0.10 would indicate that a score larger or equal to the measured one was observed in one out of ten random time courses. This distribution of Fourier scores for measured and generated random time series can be seen in Figure S4. Our model, which implies one peak per period, accounted for the vast majority of periodicity patterns. However, in rare instances, such as *rpaA* (Figure 8A), two major peaks per 24-hour period were observed, and these were usually reported as aperiodic (FDR>0.10) (e.g. *rpaA*, Table S9). Future analyses on this small subset of the genome would validate the periodicity of this interesting category of genes.

Time of peak RNA abundance was determined by two methods. The first consisted of simply identifying the sampling time point where expression was maximal during day 1 and 2. Subsequent averaging the time points leads to the peak time with a resolution of an hour. Considering the distribution derived for all probe sets, a bimodal pattern emerges (Figure S1A). Most genes peak either in the early morning hours with a maximum around 05:00 (just before lights on) or in the late evening with a maximum around 20:00 (at lights off). This approach offers a simple determination of the peak times, but it is sensitive to noise, since a single outlier measurement can interfere with the determination of peak times. The second approach to determine the time of peak expression is based on correlating a shifted cosine curve of periodicity T = 24 h with the observed expression pattern. The peak time is identified as the time shift that maximizes the correlation. By this approach, we utilized all measurement points of the time series equally for the determination of the peak time and, thus, reduced the influence of outlier measurements. Furthermore, a higher temporal resolution could be achieved (Figure S1B). Although differences were observed for some genes, the resulting distributions of peak times were similar for both approaches (Figure S1). This indicates that the influence of outlier measurements was minor in our experiment and points to a general high quality of data. The differences between both approaches can also be seen by visualizing the ordered expression matrices (data not shown). It appears that the second approach leads to a ‘smoother’ ordering of the temporal expression profiles and, thus, may be favorable in cases in which genes should be sorted according to their transcription patterns.

To obtain an estimate of the number of expressed genes measured by the microarrays, we utilized the arrays' unique feature that they included probes for *Prochlorococcus* MIT9313 and several phages besides probes for *Prochlorococcus* MED4. As we did not expect to measure expression for most of phages genes in the experiment, the corresponding phage probes sets were used for an estimation of the background intensity for non-expressed genes. First, the median signal intensity was calculated for each probe set. A crude threshold for expression was subsequently defined by determining the 0.95-quantile for signals of phages probe sets i.e. the threshold for which 95% of the phage signal intensities lie below. This threshold was chosen as we expected (and observed) that a small percentage of phage probe set will still display large expression values due to cross-hybridization with homologous genes or hybridization artifacts. The threshold obtained (29.9 arbitrary units) was then used to classify MED4 probes as “expressed” or “non-expressed.” For the following analyses, genes were included if they met one of two criteria: significant periodicity over the diel cycle (FDR<0.10), or, for the aperiodic genes (FDR≥0.10), being classified as expressed.

To examine the relative temporal expression patterns for the periodic genes, soft clustering was applied. In contrast to conventional (hard) clustering such as k-means (where genes belong to exactly one cluster), the memberships of genes to clusters were graded between 0 and 1. Large membership values imply that the genes were strongly associated with the cluster; low membership implies that the genes were poorly represented by the cluster. Soft clustering offers the advantage of producing information-rich clustering structure and of being more robust to noise [112]. For the cluster analysis, the Bioconductor package *Mfuzz* was used [113]. The clustering parameter *m* determining the ‘softness’ of the cluster was set to 1.25. The appropriate cluster number *c* was difficult to determine for this data set since there are two dominant expression patterns (corresponding to the genes peaking in the morning or evening, respectively). These two major clusters can, however, be further subdivided. Successive clustering with increasing cluster number reflected this finding showing first the main expression patterns and subsequently the minor patterns. To obtain an optimal cluster number, we assessed the functional enrichment of detected clusters varying the cluster number [30]. Consequently, the cluster number was set to 16, as it maximized the total number of subcategories of functional genes (see below) enriched for the transcriptome (data not shown).

To interpret the biological significance of the observed expression patterns, we examined the clusters obtained for enrichment of genes with known function. For this task, we utilised the functional categorization of *Prochlorococcus marinus* MED4 by the Cyanobase (http://www.kazusa.or.jp/cyano/) where 1193 genes are associated with 16 main and 62 sub-categories [114]. Of the 1193 annotated genes, 820 were found expressed in the experiment. Subsequently, we used this set of genes to associate possible functions to the expression patterns observed. The statistical significance of observing *k* genes of a defined function in a cluster with a total of *l* genes can be derived from the hyper-geometrical distribution
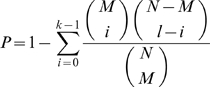
where *M* is the total number of genes attributed to the function of interest, *N* is the total number of genes annotated and *P* is the probability to observe *k* or more genes of the function of interest if they would be randomly drawn. Since multiple testing was performed, the *p*-values obtained were adjusted using the Benjamini-Hochberg procedure [115].

## Supporting Information

Table S1 Expression profiles of all MED4 probe sets. Open reading frames (“PMM####”) intergenic regions (“PMMIG…”) and non-coding RNAs (“PMM_…”) are listed in column 1, followed by annotations in column 3. PMM#### in column 1 are the annotations that were deposited in Genbank when MED4 was first sequenced [3]. Subsequently, with the sequencing of more *Prochlorococcus* strains, the genes have been renamed [2], and this new nomenclature is shown in column 2. Fourier score (column 4) and false discovery rate (FDR) for the score (column 5) are followed by calculated peak expression time (column 6) and calculated Pearson correlation with a (possibly) shifted cosine curve (column 7). Cluster assignment (column 8) and cluster membership (column 9) are followed by Cyanobase functional category (column 10) and sub-category (column 11) assignments. The final 100 columns list the mean RMA-normalized expression and the standard deviation of the mean of the 50 time points.
(4.22 MB XLS)

Table S2 (0.07 MB DOC)

Table S3 (0.10 MB DOC)

Table S4 (0.24 MB DOC)

Table S5 (0.09 MB DOC)

Table S6 (0.11 MB DOC)

Table S7 (0.07 MB DOC)

Table S8 (0.10 MB DOC)

Table S9 (0.16 MB DOC)

Table S10 (0.12 MB DOC)

Table S11 (0.06 MB DOC)

Figure S1 Histograms of the peak expression time of all periodic genes by (A) averaging over both days or (B) correlating a shifted cosine curve of periodicity T = 24 h. See text for details. Relative PAR (photosynthetically available radiation) over the experiment is represented above the histograms.
(1.36 MB EPS)

Figure S2 Members of the 16 periodic clusters (Clusters 1–16) as well as the aperiodic expressed (Cluster 17) and the unexpressed (Cluster 18) clusters. Periodic clusters were assigned and arranged chronologically, with the mean peak abundance of Cluster 1 (08:20) occurring the soonest after experimental dawn (06:00), and Cluster 16 occurring the latest (05:30). Red color indicates strong group membership (i.e high fuzziness score), while yellow indicates weak group membership. Relative PAR (photosynthetically available radiation) over the experiment is represented above the expression patterns.
(9.07 MB EPS)

Figure S3 Expression profiles of representative genes of MED4 during a pilot 12 hour light - 12 hour dark experiment, monitored by quantitative reverse transcription PCR. Values are expressed as the ratio of gene expression versus expression of the aperiodic gene *rnpB*. For all genes, including *rnpB*, and all time points, the coefficient of variation for replicate PCR reactions was less than 7.0.
(2.48 MB EPS)

Figure S4 Distribution of Fourier scores. To assess the significance of periodic expression, the distribution of Fourier scores (red line) for the measured gene expression was compared with the distribution obtained for AR(1)-based background models (blue line). The false discovery rate (orange line) denotes the fraction of Fourier scores derived from background distribution in respect to the number of scores in the observed distribution above a chosen threshold.
(0.94 MB EPS)
