# Supplementary material for: Choreography of the Transcriptome, Photophysiology, and Cell Cycle of a Minimal Photoautotroph, Prochlorococcus
Source: PLoS One. 2009 Apr 8;4(4):e5135. doi: 10.1371/journal.pone.0005135 (PMC2663038; doi:10.1371/journal.pone.0005135)
Supplement: Table S2 — (0.07 MB DOC) [file pone.0005135.s002.doc]

Table S2: Characteristics of cell division and (for comparison) cell elongation genes.

| **Process** | **PMM number** | **Gene name(s)** | **function/ gene product** | **Peak hour^a^** | **FDR for periodicity** | **Cluster** | **Cluster membership score** |
| --- | --- | --- | --- | --- | --- | --- | --- |
|  |  |  |  |  |  |  |  |
| Cell division | PMM1309 | *ftsZ* | Septal ring | 16.6 | 0.000 | 5 | 0.74 |
|  | PMM0518 | *ftsI, pbp3* | Cell wall biogenesis: cell septation | 19.2 | 0.001 | 7 | 0.56 |
|  | PMM1458 | *ftsW* | cell wall biogenesis: cell septation | 19 | 0.001 | 7 | 0.46 |
|  | PMM0616 | *amiC* | Cell wall hydrolase | 3.4 | 0.034 | 12 | 0.69 |
|  | PMM0171 | *mraW* |  | N/A | 0.139 | 18 (Undetected) | 1.00 |
|  | PMM0322 | *minC* | FtsZ septal ring placement | 17 | 0.031 | 4 | 0.53 |
|  | PMM0321 | *minD* | FtsZ septal ring placement | 16.4 | 0.000 | 4 | 0.88 |
|  | PMM0320 | *minE* | FtsZ septal ring placement | 16.8 | 0.000 | 5 | 0.87 |
|  |  |  |  |  |  |  |  |
| Cell elongation | PMM0040 | *pbp2* | cell wall biogenesis: cell elongation | 18.2 | 0.005 | 6 | 0.84 |
|  | PMM1580 | *rodA* | cell wall biogenesis: cell elongation | 22 | 0.057 | 9 | 0.56 |

^a^ h = 0, is 4 hours after the onset of dark in a 14:10 light-dark cycle.
