# Supplementary material for: Choreography of the Transcriptome, Photophysiology, and Cell Cycle of a Minimal Photoautotroph, Prochlorococcus
Source: PLoS One. 2009 Apr 8;4(4):e5135. doi: 10.1371/journal.pone.0005135 (PMC2663038; doi:10.1371/journal.pone.0005135)
Supplement: Table S3 — (0.10 MB DOC) [file pone.0005135.s003.doc]

Table S3: Characteristics of DNA synthesis genes.

| **Function** | **PMM number** | **Gene name(s)** | **Peak (hour)^a^** | **FDR for periodicity** | **Cluster** | **Cluster membership score** |
| --- | --- | --- | --- | --- | --- | --- |
| DNA repl. Initiation | PMM0565 | *dnaA* | 16 | 0.000 | 4 | 0.96 |
|  |  |  |  |  |  |  |
| DNA pol III | PMM0001 | *dnaN* | 18 | 0.000 | 5 | 0.67 |
|  | PMM0129 | *holB* | N/A | 0.196 | 18 (undetected) | 1.00 |
|  | PMM0621 | *dnaQ* | 17 | 0.000 | 5 | 0.81 |
|  | PMM0945 | *dnaE* | 17 | 0.000 | 5 | 1.00 |
|  | PMM1658 | *dnaX* | 19 | 0.005 | 6 | 0.89 |
|  |  |  |  |  |  |  |
| Primase | PMM0939 | *dnaG* | 17 | 0.000 | 5 | 0.89 |
|  |  |  |  |  |  |  |
| Helicase | PMM1674 | *dnaB* | 18 | 0.003 | 5 | 0.73 |
|  |  |  |  |  |  |  |
| SS DNA binding protein | PMM1623 | *ssb* | 17 | 0.000 | 5 | 1.00 |
|  |  |  |  |  |  |  |
| DNA ligase (NAD-binding) | PMM0659 | *ligA* | 19 | 0.001 | 6 | 0.83 |
| DNA ligase (ATP-binding) | PMM0729 | *ligB* | N/A | 0.812 | 18 (undetected) | 1.00 |
|  | PMM1679 | *ligB* | N/A | 0.137 | 17 (aperiodic) | 1.00 |
|  |  |  |  |  |  |  |
| Gyrase (subunit A) | PMM1063 | *gyrA* | 21 | 0.001 | 8 | 0.50 |
| Gyrase (subunit B) | PMM1634 | *gyrB* | 16 | 0.000 | 4 | 0.88 |
|  |  |  |  |  |  |  |
| Topoisomerase IV (Subunit A) | PMM0005 |  | 18 | 0.000 | 6 | 0.63 |
|  |  |  |  |  |  |  |
| Topoisomerase I | PMM0436 | *topA* | 20 | 0.000 | 7 | 0.98 |
|  |  |  |  |  |  |  |
| DNA pol I | PMM1140 | *polA* | 3 | 0.001 | 12 | 0.98 |

***^a^ h = 0, is 4 hours after the onset of dark in a 14:10 light-dark cycle.***
