# Supplementary material for: Choreography of the Transcriptome, Photophysiology, and Cell Cycle of a Minimal Photoautotroph, Prochlorococcus
Source: PLoS One. 2009 Apr 8;4(4):e5135. doi: 10.1371/journal.pone.0005135 (PMC2663038; doi:10.1371/journal.pone.0005135)
Supplement: Table S4 — (0.24 MB DOC) [file pone.0005135.s004.doc]

Table S4: Characteristics of electron transport and ATPase genes.

| **Function** | **PMM number** | **Gene name(s)** | **function/ gene product** | **Peak (hour)^a^** | **FDR for periodicity** | **Cluster** | **Cluster membership score** |
| --- | --- | --- | --- | --- | --- | --- | --- |
| **I. Photosynthesis** |  |  |  |  |  |  |  |
| PSI | PMM0329 | *psaE* |  | 7.4 | 0.000 | 1 | 0.99 |
|  | PMM1520 | *psaI* |  | 7.8 | 0.000 | 1 | 1.00 |
|  | PMM0469 | *psaF* |  | 8 | 0.000 | 1 | 1.00 |
|  | PMM0540 | *psaM* |  | 8 | 0.000 | 1 | 1.00 |
|  | PMM1519 | *psaL* |  | 8.2 | 0.000 | 1 | 1.00 |
|  | PMM1578 | *psaD* |  | 8.6 | 0.000 | 1 | 1.00 |
|  | PMM0468 | *psaJ* |  | 8.6 | 0.000 | 1 | 0.99 |
|  | PMM0906 | *psaK* |  | 9.4 | 0.000 | 1 | 0.93 |
|  | PMM1607 | *psaC* |  | 10.2 | 0.005 | 1 | 0.66 |
|  | PMM1523 | *psaB* | core protein | 13.8 | 0.004 | 3 | 0.92 |
|  | PMM1524 | *psaA* | core protein | 14.8 | 0.004 | 4 | 0.87 |
|  |  |  |  |  |  |  |  |
| PSII | PMM1156 | *ycf4* |  | 3.6 | 0.016 | 12 | 0.64 |
|  | PMM0507 | *psb27* |  | 5 | 0.000 | 16 | 0.91 |
|  | PMM1098 | *psbP* |  | 5.2 | 0.000 | 16 | 0.72 |
|  | PMM1152 | *ycf37* |  | 7.4 | 0.000 | 1 | 0.91 |
|  | PMM0228 | *psbO* | manganese stabilizing protein | 7.6 | 0.000 | 1 | 1.00 |
|  | PMM0926 | *psb28* |  | 7.8 | 0.000 | 1 | 0.84 |
|  | PMM0251 | *psbH* |  | 7.8 | 0.000 | 1 | 1.00 |
|  | PMM0272 | *psbK* |  | 8.2 | 0.000 | 1 | 1.00 |
|  | PMM0253 | *psbI* |  | 8.8 | 0.000 | 1 | 0.96 |
|  | PMM0317 | *psbM* |  | 9.4 | 0.000 | 2 | 0.58 |
|  | PMM0299 | *psbL* |  | 9.8 | 0.001 | 2 | 0.90 |
|  | PMM0314 | *psbT* |  | 9.8 | 0.000 | 2 | 0.62 |
|  | PMM0315 | *psbB* | CP47 | 10 | 0.000 | 1 | 0.65 |
|  | PMM0297 | *psbE* |  | 11.2 | 0.000 | 3 | 0.99 |
|  | PMM0300 | *psbJ* |  | 11.6 | 0.000 | 3 | 1.00 |
|  | PMM0298 | *psbF* |  | 11.8 | 0.000 | 3 | 1.00 |
|  | PMM0223 | *psbA* | core protein D1 | 12.2 | 0.000 | 3 | 1.00 |
|  | PMM1158 | *psbC* | CP43 | 12.4 | 0.000 | 3 | 1.00 |
|  | PMM0252 | *psbN* |  | 12.6 | 0.000 | 3 | 1.00 |
|  | PMM1157 | *psbD* | core protein D2 | 12.8 | 0.000 | 3 | 1.00 |
|  |  |  |  |  |  |  |  |
| Other PETC genes | PMM1449 | *petF* | ferrodoxin | 5.6 | 0.000 | 16 | 1.00 |
|  | PMM1171 | *isiB* | flavodoxin | 8.4 | 0.000 | 1 | 0.98 |
|  | PMM1058 | *petG* | cytochrome b6f complex | 9 | 0.012 | 2 | 0.50 |
|  | PMM0581 | *petE* | plastocyanin | 9.2 | 0.000 | 1 | 0.64 |
|  | PMM0627 | *pcb* | light harvesting complex | 11.4 | 0.003 | 3 | 0.98 |
|  | PMM0462 | *petC* | cytochrome b6f complex | 12.2 | 0.000 | 3 | 1.00 |
|  | PMM0461 | *petA* | cytochrome b6f complex, cytochrome f | 12.8 | 0.000 | 3 | 1.00 |
|  | PMM0740 | *petN* | cytochrome b6f complex | 13.8 | 0.001 | 3 | 0.96 |
|  | PMM0325 | *petB* | cytochrome b6f complex, cytochrome b6 | 15.6 | 0.000 | 4 | 1.00 |
|  | PMM0326 | *petD* | cytochrome b6f complex | 15.8 | 0.000 | 4 | 0.99 |
|  | PMM1075 | *petH* | ferrodoxin-NADP oxidoreductase (FNR) | 17 | 0.095 | 4 | 0.42 |
|  | PMM1352 | *petF* | ferrodoxin | N/A | 0.287 | 17 (Aperiodic) | 1.00 |
|  |  |  |  |  |  |  |  |
|  |  |  |  |  |  |  |  |
| **II. Respiration** |  |  |  |  |  |  |  |
| NADH dehydrogenase II | PMM0082 | *ndbB* | NDH-2 | N/A | 0.131 | 18 (Undetected) | 1.00 |
|  |  |  |  |  |  |  |  |
| NADH dehydrogenase I | PMM0435 | *ndhB* | NDH-1 subunit | 1.8 | 0.002 | 11 | 0.86 |
|  | PMM1559 | *ndhN* | NDH-1 subunit | 2.6 | 0.001 | 12 | 0.87 |
|  | PMM0594 | *ndhD* | NDH-1 subunit | 5.6 | 0.000 | 16 | 0.94 |
|  | PMM0294 | *ndhC* | NDH-1 subunit | 18 | 0.000 | 5 | 0.65 |
|  | PMM0172 | *ndhH* | NDH-1 subunit | 18 | 0.000 | 5 | 0.52 |
|  | PMM0150 | *ndhD* | NDH-1 subunit | 18.4 | 0.000 | 5 | 0.92 |
|  | PMM0145 | *ndhM* | NDH-1 subunit | 18.4 | 0.000 | 6 | 0.93 |
|  | PMM0293 | *ndhK* | NDH-1 subunit | 19.2 | 0.000 | 6 | 0.86 |
|  | PMM0160 | *ndhA* | NDH-1 subunit | 19.4 | 0.000 | 7 | 0.69 |
|  | PMM0292 | *ndhJ* | NDH-1 subunit | 19.4 | 0.001 | 7 | 0.55 |
|  | PMM0159 | *ndhI* | NDH-1 subunit | 19.6 | 0.042 | 8 | 0.59 |
|  | PMM0570 | *ndhL* | NDH-1 subunit | 19.8 | 0.001 | 7 | 0.91 |
|  | PMM0121 | *ndhO* | NDH-1 subunit | 20.2 | 0.070 | 8 | 0.41 |
|  | PMM0158 | *ndhG* | NDH-1 subunit | 20.4 | 0.010 | 7 | 0.54 |
|  | PMM0157 | *ndhE* | NDH-1 subunit | 21.4 | 0.011 | 9 | 0.71 |
|  | PMM0149 | *ndhF* | NDH-1 subunit | N/A | 0.986 | 17 (Aperiodic) | 1.00 |
|  |  |  |  |  |  |  |  |
| Cytochrome oxidase | PMM0448 | *ctaB* | cytochrome oxidase subunit | 17.2 | 0.000 | 5 | 1.00 |
|  | PMM0447 | *ctaA* | cytochrome oxidase subunit | 17.4 | 0.000 | 5 | 1.00 |
|  | PMM0444 | *ctaE* | cytochrome oxidase subunit (III) | 18 | 0.000 | 5 | 0.99 |
|  | PMM0446 | *ctaC (coxB)* | cytochrome oxidase subunit (II) | 18 | 0.000 | 5 | 1.00 |
|  | PMM0445 | *ctaD (coxA)* | cytochrome oxidase subunit (I) | 18.2 | 0.000 | 5 | 0.99 |
|  |  |  |  |  |  |  |  |
|  |  |  |  |  |  |  |  |
| **III. Proton-translocating ATPase** | |  |  |  |  |  |  |
|  | PMM1453 | *atpF* | B/B' subunit | 5.2 | 0.000 | 16 | 1.00 |
|  | PMM1452 | *atpH* | delta subunit | 5.2 | 0.000 | 16 | 1.00 |
|  | PMM1454 | *atpG* | B/B' subunit | 5.4 | 0.000 | 16 | 1.00 |
|  | PMM1455 | *atpK* | C subunit | 5.4 | 0.001 | 16 | 1.00 |
|  | PMM1456 | *atpI* | A subunit | 5.6 | 0.000 | 16 | 1.00 |
|  | PMM1451 | *atpA* | alpha subunit | 5.8 | 0.000 | 16 | 1.00 |
|  | PMM1438 | *atpB* | beta subunit | 6 | 0.000 | 16 | 1.00 |
|  | PMM1450 | *atpC* | gamma subunit | 6 | 0.000 | 16 | 1.00 |
|  | PMM1439 | *atpE* | epsilon subunit | 6 | 0.000 | 16 | 1.00 |

***^a^ h = 0, is 4 hours after the onset of dark in a 14:10 light-dark cycle.***
