# Supplementary material for: Choreography of the Transcriptome, Photophysiology, and Cell Cycle of a Minimal Photoautotroph, Prochlorococcus
Source: PLoS One. 2009 Apr 8;4(4):e5135. doi: 10.1371/journal.pone.0005135 (PMC2663038; doi:10.1371/journal.pone.0005135)
Supplement: Table S5 — (0.09 MB DOC) [file pone.0005135.s005.doc]

Table S5: Characteristics of the high-light inducible proteins (HLIPs).

| **PMM Number** | **Gene name(s)** | **Peak (hour)^a^** | **FDR for periodicity** | **Cluster** | **Cluster membership score** | **Copy number** |
| --- | --- | --- | --- | --- | --- | --- |
|  |  |  |  |  |  |  |
| PMM1397/PMM0816^b^ | *hli08 / hli18* | 1.2 | 0.074 | 10 | 0.65 | multi |
| PMM1398/PMM0817^b^ | *hli07 / hli17* | 1.4 | 0.093 | 10 | 0.60 | multi |
| PMM1396/PMM0815^b^ | *hli09 / hli19* | 1.4 | 0.093 | 10 | 0.49 | multi |
| PMM0689 | *hli22* | 1.4 | 0.038 | 11 | 0.63 | multi |
| PMM1118 | *hli04* | 1.6 | 0.001 | 10 | 0.52 | multi |
| PMM1399/PMM0818^b^ | *hli06 / hli16* | 1.8 | 0.089 | 10 | 0.40 | multi |
| PMM1135 | *hli14* | 1.8 | 0.020 | 10 | 0.41 | multi |
| PMM0064 | *hli02* | 3.6 | 0.001 | 12 | 0.69 | single |
| PMM0093 | *hli01* | 8 | 0.001 | 1 | 0.96 | single |
| PMM1317 | *hli13* | 12.6 | 0.001 | 3 | 1.00 | single^c^ |
| PMM1390 | *hli10* | 14.8 | 0.000 | 4 | 0.99 | multi |
| PMM0471 | *hli20* | 16.6 | 0.000 | 5 | 0.69 | single |
| PMM1385 | *hli11* | 21.6 | 0.000 | 8 | 0.82 | multi |
| PMM1384 | *hli12* | 22 | 0.001 | 8 | 0.83 | multi |
| PMM1482 | *hli03* | 22.2 | 0.011 | 9 | 0.72 | single |
| PMM1404 | *hli05* | N/A | 0.155 | 17 (Aperiodic) | 1.00 | multi |
| PMM1128 | *hli15* | N/A | 0.734 | 17 (Aperiodic) | 1.00 | multi |
| PMM0690 | *hli21* | N/A | 0.745 | 17 (Aperiodic) | 1.00 | multi |

***^a^ h = 0, is 4 hours after the onset of dark in a 14:10 light-dark cycle.***

***^b^ Two identical copies are present in the MED4 genome.***

***^c^ Single copy in many but not all marine cyanobacteria.***
