# Supplementary material for: Choreography of the Transcriptome, Photophysiology, and Cell Cycle of a Minimal Photoautotroph, Prochlorococcus
Source: PLoS One. 2009 Apr 8;4(4):e5135. doi: 10.1371/journal.pone.0005135 (PMC2663038; doi:10.1371/journal.pone.0005135)
Supplement: Table S6 — (0.11 MB DOC) [file pone.0005135.s006.doc]

Table S6: Characteristics of the carbon metabolism genes.

| **Pathway** | **PMM number** | **Gene name(s)** | **function/ gene product** | **Peak (hour)^a^** | **FDR for periodicity** | **Cluster** | **Cluster membership score** |
| --- | --- | --- | --- | --- | --- | --- | --- |
|  |  |  |  |  |  |  |  |
| **Calvin Cycle, glycogen synthesis** | PMM0584 | *glgB* | 1,4-alpha-glucan branching enzyme | 4.8 | 0.000 | 16 | 0.94 |
|  | PMM0609 | *glgA* | ADPglucose--glucosyltransferase | 4.8 | 0.000 | 15 | 0.56 |
|  | PMM0769 | *glgC* | ADP-glucose pyrophosphorylase | 5.2 | 0.000 | 16 | 0.98 |
|  | PMM0549 | *csoS1* | carboxysome | 5.2 | 0.000 | 16 | 0.99 |
|  | PMM0829 | *tpi, cbbJ* | Triosephosphate isomerase | 5.4 | 0.000 | 16 | 0.98 |
|  | PMM0554 | *ccmI, orfA* | carboxysome, putative peptide A | 5.4 | 0.039 | 15 | 0.43 |
|  | PMM0781 | *cbbA, cfxA, fbaA, fda* | Fructose-bisphosphate/sedoheptulose-1,7-bisphosphate aldolase | 5.6 | 0.000 | 16 | 0.99 |
|  | PMM0767 | *glpX, cbbF, fbp* | Fructose-1,6-bisphosphatase/sedoheptulose-1,7-bisphosphatase | 5.8 | 0.001 | 16 | 0.98 |
|  | PMM0550 | *rbcL, cbbL* | carboxysome: Rubisco large chain | 5.8 | 0.000 | 16 | 0.99 |
|  | PMM0552 | *csoS2* | carboxysome | 5.8 | 0.000 | 16 | 0.95 |
|  | PMM0555 | *ccmI, orfB* | carboxysome, putative peptide B | 5.8 | 0.009 | 15 | 0.40 |
|  | PMM0785 | *prk, cbbP* | Phosphoribulokinase | 6 | 0.000 | 16 | 1.00 |
|  | PMM0551 | *rbcS, cbbS* | carboxysome: Rubisco small chain | 6 | 0.000 | 16 | 0.98 |
|  | PMM0553 | *csoS3* | carboxysome: Carbonic anhydrase | 6 | 0.000 | 16 | 0.84 |
|  | PMM0023 | *gap2* | Glyceraldehyde 3-phosphatedehydrogenase | 6.8 | 0.001 | 16 | 0.56 |
|  | PMM0195 | *pgk, cbbK* | Phosphoglycerate kinase | 9.4 | 0.000 | 2 | 0.88 |
|  |  |  |  |  |  |  |  |
|  |  |  |  |  |  |  |  |
|  |  |  |  |  |  |  |  |
| **Pentose phosphate pathway, glycogen degradation** | PMM1322 | *glgX* | Glycogen branching enzyme | 16.6 | 0.000 | 6 | 0.98 |
|  | PMM0519 | *tal* | Transaldolase | 17.6 | 0.000 | 5 | 1.00 |
|  | PMM1601 | *glgP* | Glycogen phosphorylase | 17.6 | 0.000 | 5 | 1.00 |
|  | PMM0770 | *gnd* | 6-phosphogluconate dehydrogenase | 18 | 0.000 | 5 | 0.98 |
|  | PMM0771 | *devB, pgl* | 6-phosphogluconolactonase | 18.2 | 0.000 | 6 | 0.84 |
|  | PMM1074 | *zwf* | Glucose-6-phosphate dehydrogenase | 18.8 | 0.000 | 6 | 0.94 |
|  |  |  |  |  |  |  |  |
|  |  |  |  |  |  |  |  |
| **Shared use** | PMM1489 | *rpiA, cbbI* | Ribose 5-phosphate isomerase | 4.2 | 0.000 | 13 | 1.00 |
|  | PMM0766 | *rpe, cbbE* | Ribulose-phosphate 3-epimerase | 5 | 0.000 | 16 | 0.99 |
|  | PMM1610 | *tktA, cbbT* | Transketolase | 5.4 | 0.000 | 16 | 1.00 |
|  | PMM0076 | *pgm* | Phosphoglucomutase | 9.4 | 0.000 | 2 | 0.99 |
|  | PMM0890 | *pgi* | Phosphoglucose isomerase | 15.8 | 0.007 | 4 | 0.93 |

***^a^ h = 0, is 4 hours after the onset of dark in a 14:10 light-dark cycle.***
