# Supplementary material for: Choreography of the Transcriptome, Photophysiology, and Cell Cycle of a Minimal Photoautotroph, Prochlorococcus
Source: PLoS One. 2009 Apr 8;4(4):e5135. doi: 10.1371/journal.pone.0005135 (PMC2663038; doi:10.1371/journal.pone.0005135)
Supplement: Table S7 — (0.07 MB DOC) [file pone.0005135.s007.doc]

Table S7: Characterization of the phosphorus metabolism genes.

| **Categories** | **PMM number** | **Gene name(s)** | **function/ gene product** | **Peak (hour)^a^** | **FDR for periodicity** | **Cluster** | **Cluster membership score** |
| --- | --- | --- | --- | --- | --- | --- | --- |
|  |  |  |  |  |  |  |  |
| Regulation | PMM0705 | *phoB* | two-component response regulator | 19.2 | 0.005 | 6 | 0.80 |
|  | PMM0706 | *phoR* | two-component sensor histidine kinase | 21.8 | 0.343 | 18 | 1.00 |
|  |  |  |  |  |  |  |  |
| Phosphate uptake | PMM0709 | *phoE* | porin | 21.6 | 0.000 | 9 | 0.91 |
|  | PMM0710 | *pstS* | ABC transporter, substrate binding protein | 3.6 | 0.030 | 13 | 0.67 |
|  | PMM0723 | *pstC* | ABC transporter, permease component | 19.8 | 0.000 | 7 | 0.79 |
|  | PMM0724 | *pstA* | ABC transporter, permease component | 21.6 | 0.004 | 9 | 0.80 |
|  | PMM0725 | *pstB* | ABC transporter, ATP binding subunit | 20.8 | 0.000 | 7 | 0.77 |
|  |  |  |  |  |  |  |  |
| Organic phosphate conversion | PMM0708 | *phoA* | alkaline phosphatase | 21.8 | 0.004 | 8 | 0.53 |
|  | PMM1624 | *dedA* | alkaline phosphatase-like protein | 14.4 | 0.026 | 4 | 0.72 |

^a^ h = 0, is 4 hours after the onset of dark in a 14:10 light-dark cycle.
