# Supplementary material for: Choreography of the Transcriptome, Photophysiology, and Cell Cycle of a Minimal Photoautotroph, Prochlorococcus
Source: PLoS One. 2009 Apr 8;4(4):e5135. doi: 10.1371/journal.pone.0005135 (PMC2663038; doi:10.1371/journal.pone.0005135)
Supplement: Table S8 — (0.10 MB DOC) [file pone.0005135.s008.doc]

Table S8: Characteristics of the nitrogen metabolism genes.

| **Categories** | **PMM number** | **Gene name(s)** | **function/ gene product** | **Peak (hour)^a^** | **FDR for periodicity** | **Cluster** | **Cluster membership score** |
| --- | --- | --- | --- | --- | --- | --- | --- |
| Regulation | PMM0246 | *ntcA* | Global nitrogen regulatory protein | N/A | 0.647 | 18 (Undetected) | 1.00 |
|  | PMM0393 | *pipX* |  | 17.8 | 0.000 | 5 | 0.97 |
|  | PMM1463 | *glnB* | Nitrogen regulatory protein P-II | 9.4 | 0.054 | 1 | 0.64 |
|  |  |  |  |  |  |  |  |
| Ammonium assimilation | PMM0920 | *glnA* | Glutamine synthetase, glutamate--ammonia ligase | 16.4 | 0.000 | 4 | 0.81 |
|  | PMM1512 | *glsF* | Ferredoxin-dependent glutamate synthase,Fd-GOGAT | 19 | 0.000 | 6 | 0.95 |
|  | PMM1596 | *icd* | Isocitrate dehydrogenase | 17 | 0.000 | 5 | 0.75 |
|  |  |  |  |  |  |  |  |
| Ammonium uptake | PMM0263 | *amt1* | Ammonium transporter | 19.2 | 0.000 | 6 | 0.36 |
|  |  |  |  |  |  |  |  |
| Oligopeptide uptake | PMM1049 |  | oligopeptide ABC transporter, substrate binding protein | 20 | 0.000 | 7 | 0.92 |
|  | PMM0421 |  | putative ABC transporter, oligopeptides | 18.8 | 0.000 | 6 | 0.93 |
|  |  |  |  |  |  |  |  |
| Urea uptake | PMM0970 | *urtA* | Urea ABC transporter, substrate binding protein | 9.6 | 0.007 | 1 | 0.61 |
|  | PMM0971 | *urtB* | Urea ABC transporter | 8.6 | 0.044 | 1 | 0.85 |
|  | PMM0972 | *urtC* | Urea ABC transporter, membrane protein | N/A | 0.261 | 17 (Aperiodic) | 1.00 |
|  | PMM0973 | *urtD* | Urea ABC Transporter, ATP binding subunit | 5.6 | 0.053 | 15 | 0.46 |
|  | PMM0974 | *urtE* | Urea ABC Transporter, ATP binding subunit | 3.6 | 0.054 | 12 | 0.67 |
| Urea conversion to ammonium | PMM0965 | *ureA* | Urease gamma subunit | 19.4 | 0.000 | 7 | 0.62 |
|  | PMM0964 | *ureB* | Urease beta subunit | 20.2 | 0.000 | 7 | 0.92 |
|  | PMM0963 | *ureC* | Urease alpha subunit | 20.6 | 0.000 | 7 | 0.86 |
|  | PMM0966 | *ureD* | Urease accessory protein | 22.6 | 0.056 | 9 | 0.42 |
|  | PMM0967 | *ureE* | Urease accessory protein | N/A | 0.722 | 18 (Undetected) | 1.00 |
|  | PMM0968 | *ureF* | Urease accessory protein | N/A | 0.297 | 18 (Undetected) | 1.00 |
|  | PMM0969 | *ureG* | Urease accessory protein | 19.8 | 0.000 | 7 | 0.94 |
|  |  |  |  |  |  |  |  |
| Cyanate uptake | PMM0370 | *cynA* | Cyanate ABC transporter, substrate binding protein | 10.6 | 0.051 | 3 | 0.64 |
|  | PMM0371 |  | Cyanate ABC transporter | 11 | 0.269 | 17 (Aperiodic) | 1.00 |
|  | PMM0372 |  | Cyanate ABC transporter | 12.4 | 0.308 | 18 (Undetected) | 1.00 |
| Cyanate conversion to ammonium | PMM0373 | *cynS* | Cyanate lyase | 11.2 | 0.019 | 3 | 0.91 |

***^a^ h = 0, is 4 hours after the onset of dark in a 14:10 light-dark cycle.***
