# Supplementary material for: Choreography of the Transcriptome, Photophysiology, and Cell Cycle of a Minimal Photoautotroph, Prochlorococcus
Source: PLoS One. 2009 Apr 8;4(4):e5135. doi: 10.1371/journal.pone.0005135 (PMC2663038; doi:10.1371/journal.pone.0005135)
Supplement: Table S9 — (0.16 MB DOC) [file pone.0005135.s009.doc]

Table S9: Characteristics of the regulatory proteins.

| **Class** | **PMM number** | **Gene name(s)** | **Peak (hour)^a^** | **FDR for periodicity** | **Cluster** | **Cluster membership score** |
| --- | --- | --- | --- | --- | --- | --- |
|  |  |  |  |  |  |  |
| Sigma factors | PMM1629 |  | 6 | 0.000 | 15 | 0.54 |
|  | PMM1289 |  | 14 | 0.000 | 4 | 0.97 |
|  | PMM1697 |  | 15 | 0.003 | 5 | 0.92 |
|  | PMM0577 |  | 17 | 0.000 | 4 | 0.52 |
|  | PMM0496 | *rpoD* | 22 | 0.003 | 10 | 0.45 |
|  |  |  |  |  |  |  |
| Sensor kinase | PMM0269 | *hik01* | 20 | 0.003 | 7 | 0.84 |
|  | PMM1077 | *hik02, sasA* | 21 | 0.062 | 9 | 0.71 |
|  | PMM1341 | *nblS, hik04* | 23 | 0.067 | 10 | 0.95 |
|  | PMM1579 | *hik05* | 23 | 0.043 | 10 | 0.91 |
|  | PMM0706 | *phoR, hik03* | N/A | 0.343 | 18 (Undetected) | 1.00 |
|  |  |  |  |  |  |  |
| Response regulators | PMM0134 | *rpaB, rer02* | 3 | 0.000 | 13 | 0.96 |
|  | PMM1113 | *rer05* | 10 | 0.000 | 1 | 0.67 |
|  | PMM0705 | *phoB, rer06* | 18 | 0.005 | 6 | 0.80 |
|  | PMM0169 | *rer03* | 23 | 0.051 | 8 | 0.78 |
|  | PMM0128 | *rpaA, rer01* | N/A | 0.141 | 17 (Aperiodic) | 1.00 |
|  | PMM1619 | *rer04* | N/A | 0.279 | 17 (Aperiodic) | 1.00 |
|  |  |  |  |  |  |  |
| Circadian clock | PMM1343 | *kaiB* | 4 | 0.000 | 15 | 0.86 |
|  | PMM1342 | *kaiC* | 22 | 0.062 | 9 | 0.74 |
|  |  |  |  |  |  |  |
| Helix-turn-helix | PMM1637 |  | 2 | 0.040 | 12 | 0.86 |
|  | PMM1082 |  | 3 | 0.018 | 10 | 0.76 |
|  | PMM1176 |  | 6 | 0.000 | 15 | 0.90 |
|  | PMM0154 |  | 14 | 0.009 | 4 | 0.78 |
|  | PMM0509 |  | 16 | 0.082 | 6 | 0.53 |
|  | PMM0734 |  | 18 | 0.048 | 8 | 0.44 |
|  | PMM0988 |  | 18 | 0.000 | 6 | 0.52 |
|  | PMM1391 |  | 18 | 0.001 | 6 | 0.80 |
|  |  |  |  |  |  |  |
| *fur*-like | PMM0637 | *fur* | 19 | 0.001 | 6 | 0.60 |
|  | PMM1030 |  | N/A | 0.275 | 17 (Aperiodic) | 1.00 |
|  |  |  |  |  |  |  |
| *crp*-like | PMM0718 |  | N/A | 0.152 | 18 (Undetected) | 1.00 |
|  | PMM0806 |  | N/A | 0.214 | 17 (Aperiodic) | 1.00 |
|  |  |  |  |  |  |  |
| N regulation | PMM1463 | *glnB, PII* | 12 | 0.054 | 1 | 0.64 |
|  | PMM0393 | *pipX* | 18 | 0.000 | 5 | 0.97 |
|  | PMM0246 | *ntcA* | N/A | 0.647 | 18 (Undetected) | 1.00 |
|  |  |  |  |  |  |  |
| Others | PMM0147 | *rbcR, cbbR, lysR* | 1 | 0.005 | 10 | 0.49 |
|  | PMM1278 | *cpmA* | 1 | 0.078 | 11 | 0.42 |
|  | PMM0363 |  | 3 | 0.021 | 12 | 0.82 |
|  | PMM0679 |  | 3 | 0.000 | 15 | 0.97 |
|  | PMM0684 |  | 18 | 0.027 | 6 | 0.63 |
|  | PMM0939 |  | 16 | 0.000 | 5 | 0.89 |
|  | PMM1125 |  | 5 | 0.004 | 15 | 0.49 |
|  | PMM1369 |  | 9 | 0.000 | 1 | 0.67 |
|  | PMM1642 |  | 15 | 0.023 | 6 | 0.59 |
|  | PMM0262 | *sfsA* | 16 | 0.009 | 6 | 0.81 |
|  | PMM1262 | *lexA* | 16 | 0.000 | 5 | 0.96 |
|  | PMM0565 | *dnaA* | 17 | 0.000 | 1 | 0.96 |
|  | PMM1393 |  | 19 | 0.059 | 8 | 0.39 |
|  | PMM0714 |  | 23 | 0.035 | 8 | 0.75 |

***^a^ h = 0, is 4 hours after the onset of dark in a 14:10 light-dark cycle.***
