# Supplementary material for: Choreography of the Transcriptome, Photophysiology, and Cell Cycle of a Minimal Photoautotroph, Prochlorococcus
Source: PLoS One. 2009 Apr 8;4(4):e5135. doi: 10.1371/journal.pone.0005135 (PMC2663038; doi:10.1371/journal.pone.0005135)
Supplement: Table S10 — (0.12 MB DOC) [file pone.0005135.s010.doc]

Table S10: Genes with higher or lower expression in continuous light versus the mean expression under a light-dark cycle.

| | **Gene** | **Function** | **Continuous: mean diel expression** | **q-value** | | --- | --- | --- | --- | |  |  |  |  | | PMM0818 | hli16 possible high light inducible protein | 7.75 | 7.70E-03 | | PMM0347 | conserved hypothetical | 7.19 | 1.09E-05 | | PMM0348 | possible Spectrin repeat | 6.94 | 4.42E-04 | | PMM0817 | hli17 possible high light inducible protein | 6.49 | 5.37E-03 | | PMM1396 | hli9 possible high light inducible protein | 6.25 | 1.85E-03 | | PMM0861 | possible Virion host shutoff protein | 6.10 | 1.12E-02 | | PMM1397 | hli8 possible high light inducible protein | 5.99 | 1.74E-03 | | PMM1135 | hli14 possible high light inducible protein | 5.43 | 2.87E-03 | | PMM1629 | Type II alternative RNA polymerase sigma factor, sigma-70 family | 4.95 | 7.36E-05 | | PMM1400 | possible Hemagglutinin-neuraminidase | 4.83 | 2.07E-03 | | PMM1402 | Conserved hypothetical protein | 4.48 | 1.88E-05 | | PMM1390 | hli10 possible high light inducible protein | 4.37 | 7.73E-04 | | PMM1704 | dnaK2 Molecular chaperone DnaK2, heat shock protein hsp70-2 | 4.22 | 7.72E-05 | | PMM0699 | conserved hypothetical | 4.17 | 2.71E-05 | | PMM1365 | possible MATH domain | 4.12 | 7.84E-06 | | PMM0901 | htpG heat shock protein HtpG | 3.68 | 2.81E-06 | | PMM0700 | conserved hypothetical protein | 3.53 | 4.16E-05 | | PMM1052 | SufE protein probably involved in Fe-S center assembly | 3.37 | 1.99E-03 | | PMM1028 | conserved hypothetical | 3.30 | 1.19E-04 | | PMM0452 | groL GroEL2 protein (Chaperonin cpn60 2) | 2.92 | 4.99E-05 | | PMM1283 | Integral membrane protein, interacts with FtsH | 2.64 | 4.84E-03 | | PMM0577 | Putative type II alternative sigma factor, sigma70 family | 2.55 | 1.49E-04 | | PMM1118 | hli4 possible high light inducible protein | 2.53 | 3.77E-05 | | PMM1150 | putative thioredoxin reductase | 2.52 | 5.68E-05 | | PMM0016 | grpE Heat shock protein GrpE | 2.51 | 1.18E-05 | | PMM1385 | hli11 possible high light inducible protein | 2.36 | 3.95E-04 | | PMM1405 | hypothetical | 2.35 | 4.51E-05 | | PMM1118 | hli4 possible high light inducible protein | 2.23 | 2.92E-06 | | PMM0407 | cysK1 O-acetylserine (thiol)-lyase A | 2.23 | 8.61E-06 | | PMM0958 | conserved hypothetical | 2.23 | 1.30E-04 | | PMM1289 | Type II alternative RNA polymerase sigma factor, sigma-70 family | 2.19 | 2.33E-03 | | PMM1611 | thiC ThiC family | 2.15 | 5.56E-04 | | PMM1264 | ftsH3 cell division protein FtsH3 | 2.14 | 3.12E-05 | | PMM0321 | minD putative septum site-determining protein MinD | 2.12 | 8.71E-05 | | PMM1462 | conserved hypothetical protein | 2.06 | 1.58E-02 | | PMM0690 | hli21 possible high light inducible protein | 2.04 | 2.45E-05 | | PMM1528 | HNH endonuclease family protein | 2.03 | 3.06E-04 | | PMM0043 | flavoprotein | 2.03 | 1.08E-03 | | PMM1437 | groES GroES protein (Chaperonin cpn10) | 2.01 | 1.18E-05 | |  |  |  |  | | PMM0087 | conserved hypothetical protein | 0.28 | 7.76E-04 | | PMM1672 | des9 Fatty acid desaturase, type 1 | 0.32 | 2.81E-06 | | PMM1079 | possible Villin headpiece domain | 0.37 | 2.81E-06 | | PMM0305 | cpeB Phycobilisome protein | 0.38 | 2.13E-04 | | PMM1485 | rpoB RNA polymerase beta subunit | 0.39 | 8.84E-04 | | PMM0227 | cysD ATP-sulfurylase | 0.39 | 2.92E-06 | | PMM0751 | conserved hypothetical protein | 0.42 | 2.21E-05 | | PMM0768 | hemA glutamyl-tRNA reductase | 0.43 | 1.09E-03 | | PMM1609 | fabF 3-oxoacyl-[acyl-carrier-protein] synthase II | 0.45 | 5.99E-04 | | PMM0088 | conserved hypothetical protein | 0.46 | 2.81E-06 | | PMM0245 | cob(I)alamin adenosyltransferase | 0.46 | 9.40E-05 | | PMM0399 | Putative deoxyribose-phosphate aldolase | 0.46 | 3.56E-04 | | PMM1501 | S1 RNA binding domain:Ribonuclease E and G | 0.47 | 8.89E-05 | | PMM0496 | sigA, rpoD Putative principal RNA polymerase sigma factor | 0.47 | 2.82E-05 | | PMM0056 | conserved hypothetical protein | 0.48 | 6.40E-05 | | PMM0643 | metA putative homoserine O-succinyltransferase | 0.48 | 3.32E-03 | | PMM1228 | hypothetical protein | 0.50 | 7.92E-05 | |
| --- | --- | --- | --- | --- | --- | --- | --- | --- | --- | --- | --- | --- | --- | --- | --- | --- | --- | --- | --- | --- | --- | --- | --- | --- | --- | --- | --- | --- | --- | --- | --- | --- | --- | --- | --- | --- | --- | --- | --- | --- | --- | --- | --- | --- | --- | --- | --- | --- | --- | --- | --- | --- | --- | --- | --- | --- | --- | --- | --- | --- | --- | --- | --- | --- | --- | --- | --- | --- | --- | --- | --- | --- | --- | --- | --- | --- | --- | --- | --- | --- | --- | --- | --- | --- | --- | --- | --- | --- | --- | --- | --- | --- | --- | --- | --- | --- | --- | --- | --- | --- | --- | --- | --- | --- | --- | --- | --- | --- | --- | --- | --- | --- | --- | --- | --- | --- | --- | --- | --- | --- | --- | --- | --- | --- | --- | --- | --- | --- | --- | --- | --- | --- | --- | --- | --- | --- | --- | --- | --- | --- | --- | --- | --- | --- | --- | --- | --- | --- | --- | --- | --- | --- | --- | --- | --- | --- | --- | --- | --- | --- | --- | --- | --- | --- | --- | --- | --- | --- | --- | --- | --- | --- | --- | --- | --- | --- | --- | --- | --- | --- | --- | --- | --- | --- | --- | --- | --- | --- | --- | --- | --- | --- | --- | --- | --- | --- | --- | --- | --- | --- | --- | --- | --- | --- | --- | --- | --- | --- | --- | --- | --- | --- | --- | --- | --- | --- | --- | --- | --- | --- | --- | --- | --- | --- | --- | --- | --- | --- | --- | --- | --- | --- | --- | --- | --- | --- |
