# Supplementary material for: Choreography of the Transcriptome, Photophysiology, and Cell Cycle of a Minimal Photoautotroph, Prochlorococcus
Source: PLoS One. 2009 Apr 8;4(4):e5135. doi: 10.1371/journal.pone.0005135 (PMC2663038; doi:10.1371/journal.pone.0005135)
Supplement: Table S11 — (0.06 MB DOC) [file pone.0005135.s011.doc]

Table S11: Quantitative reverse-transcription-PCR data and primers used.

| Gene | Time of Max expression | Time of Min expression | Max/Min  expression | Forward primer (5’-> 3’) | Reverse primer (5’-> 3’) |
| --- | --- | --- | --- | --- | --- |
| *ftsZ* | 12:00 | 08:00 | 19 | ftsZ-675F: AATGACTGAAGCTGGCACTGC | ftsZ-765R: ACTATTCATTGCGGCTTGAGC |
| *dnaA* | 12:00 | 00:00 | 9.4 | dnaA-434F: CAGCTTTAGCAGTGGCAGAA | dnaA-538R: AATGACCAACAGCTTGCATC |
| *psbA* | 12:00 | 00:00 | 14 | psbA-26F: CTTCGCTGTTAAAAGGCTGGC | psbA-114R: CATTAAGACGCCGAACCAACC |
| *pcb* | 12:00 | 00:00 | 2.6 | pcb-90F: TCATGTCGCTCATGCAGGG | pcb-181R: GACCCATTGGGACACTGGG |
| *rbcL* | 04:00 | 16:00 | 53 | rbcL-55F: CCTGAATATGTCCCCCTCGA | rbcL-145R: CCGCTGCAACTTCTTCT |
| *kaiC* | 20:00 | 12:00 | 3.7 | kaiC-730F: GCCTTAGGAGCGATGAGATT | kaiC-825R: AAAATAACCCCCTCCACACA |
| *rpoD* | 20:00 | 12:00 | 17 | rpoD-591F AATCAGAGCTGCCGAAAAAT | rpoD-692R TGATCTGCTATCGCTCGTGT |
| *rnpB* | N/A | N/A | N/A | rnpB-1F: TTGAGGAAAGTCCGGGCTC | rnpB-91R: GCGGTATGTTTCTGTGGCACT |
